# Multiple BnaSOBIR1-Associated Receptor-like Proteins Contribute to Enhanced Resistance to *Sclerotinia sclerotiorum* in *Brassica napus* L. (Oilseed Rape)

**DOI:** 10.3390/plants15162545

**Published:** 2026-08-21

**Authors:** Chenghuizi Yang, Liying Ma, Jieying Nong, Shitou Xia

**Affiliations:** 1 Department of Agriculture and Forestry, Hainan Tropical Ocean University, Sanya 572022, China; ychz121@hntou.edu.cn (C.Y.); liyingma@hntou.edu.cn (L.M.); 2 Hunan Provincial Key Laboratory of Phytohormones and Growth Development, College of Bioscience and Biotechnology, Hunan Agricultural University, Changsha 410128, China; njy@stu.hunau.edu.cn

**Keywords:** *Brassica napus*, BnaSOBIR1-associated receptor-like proteins, *Sclerotinia sclerotiorum*, Ssnlp24*_Ss_*_NEP2_, immune receptor complexes

## Abstract

As a major fungal pathogen of *Brassica napus*, *Sclerotinia sclerotiorum* causes significant yield reductions worldwide. Receptor-like proteins (RLPs) are essential components of plant immunity, but the functions of many RLPs in *B. napus* still remain unclear. In this study, three BnaSOBIR1-interacting leucine-rich repeat RLPs (LRR-RLPs), named BnaRLP-G13-2, BnaRLP-G13-3, and BnaRLP-G13-4, were identified by yeast two-hybrid (Y2H) and bimolecular fluorescence complementation (BiFC) assays. Promoter analysis revealed diverse cis-acting elements involved in stress and phytohormone responses. Overexpression of these genes significantly improved resistance to *S. sclerotiorum* in both *Arabidopsis thaliana* and *B. napus*. Consistently, BnaRLP-G13-2/3/4 restored disease resistance and NLP-induced ROS production in the *rlp23-1* mutant. Furthermore, BiFC assays suggested an association between BnaRLP-G13-2/3/4 and Ssnlp24_SsNEP2_-associated perception, although direct biochemical binding remains to be demonstrated. These findings advance the understanding of BnaSOBIR1-associated BnaRLP-G13-2/3/4-mediated immunity and provide new insights into enhancing disease resistance in oilseed crops.

## 1. Introduction

To withstand diverse pathogen challenges, plants have evolved a two-tiered innate immune system consisting of pattern-triggered immunity (PTI) and effector-triggered immunity (ETI). These two immune layers are interconnected at the signaling level and mutually reinforce each other to activate effective defense responses [1,2,3,4]. PTI is activated upon the perception of pathogen- and damage-associated molecular patterns by pattern recognition receptors (PRRs). Plant PRRs mainly include receptor-like kinases (RLKs) and receptor-like proteins (RLPs). However, RLPs lack intracellular kinase domains and are therefore unable to transmit immune signals to downstream components on their own [5]. By contrast, RLKs are major components of PTI signaling. Many RLKs interact with RLPs and recruit SOMATIC EMBRYOGENESIS RECEPTOR KINASE (SERK) family members (such as BAK1) to assemble functional receptor complexes. Activation of these complexes initiates downstream immune signaling events, including MAP kinase cascades, Ca^2+^ influx, ROS production, and defense-related transcriptional changes [6,7]. These early immune signaling events are further integrated with phytohormone pathways, particularly those mediated by salicylic acid (SA), jasmonic acid (JA), and ethylene (ET), which play crucial roles in coordinating defense responses against biotrophic and necrotrophic pathogens [8,9].

As an important member of the RLKs, SUPPRESSOR OF BIR1-1 (SOBIR1) forms complexes with multiple immunity-related leucine-rich repeat RLPs (such as SlCf-4, SlVe1, AtRLP23, AtRLP30, AtRLP42, and RXEG1), thereby maintaining RLP protein stability and plasma membrane localization and providing the essential kinase activity required for PTI signal activation [10,11,12,13,14]. Upon recognition of PAMPs or DAMPs by these receptor complexes, the RLP-SOBIR1 complex further recruits SERK family co-receptors (predominantly BAK1), to form an activated receptor complex that undergoes trans-phosphorylation and initiates downstream immune signaling cascades [15]. In addition, SOBIR1 interacts with receptor-like cytoplasmic kinases (RLCKs) to fine-tune the strength and duration of RLP-mediated immune signaling [16,17]. Notably, SOBIR1 is also involved in the ETI pathway through ligand-independent association with the core components EDS1-PAD4-ADR1, in a ligand-independent manner, thereby providing a mechanistic link between PTI and ETI signaling [16]. SOBIR1-mediated immunity, however, remains to be further elucidated and harnessed for improving plant resistance to pathogens.

The LRR-RLP member *At*RLP23 recognizes the conserved nlp24 peptide derived from necrosis- and ethylene-inducing proteins (NLPs), which are widely distributed among many pathogens. This recognition activates plant immune responses through the assembly of an RLP23-SOBIR1-BAK1 ternary receptor complex [12]. However, nlp24 peptides from different pathogens are not equally recognized by AtRLP23 [18]. Heterologous expression of *AtRLP23* in potato [12], popla [19] and tomato [20] enhances broad-spectrum resistance to multiple pathogens. The C-terminal domain of RLPs is essential for effective immune signaling in heterologous expression systems [20]. Sclerotinia stem rot caused by *Sclerotinia sclerotiorum* leads to severe yield losses in *Brassica napus*. The AtRLP23 homologs BnRLP26, BnRLP41, and BnaRLP-G13-1 enhance resistance against Sclerotinia stem rot by recognizing conserved NLP-derived peptides from *S. sclerotiorum* [21,22]. In addition, the LRR-RLPs RLP30 and RXEG1 can also form complexes with SOBIR1 and BAK1, thereby conferring resistance to *S. sclerotiorum* [13,23]. Given that *B. napus* is an allotetraploid species, whether additional LRR-RLP members exist that contribute to enhanced resistance against Sclerotinia stem rot remains an important question for further investigation.

In this study, we performed an interaction-based screening in *B. napus* using the previously identified BnaSOBIR1-1 and BnaSOBIR1-2 [22], which led to the identification of three novel LRR-RLP members. These receptors are associated with Ssnlp24_SsNEP2_-triggered immune responses and, when overexpressed, enhance resistance against Sclerotinia stem rot in *B. napus*. Moreover, all three LRR-RLPs are able to functionally complement the immune defects caused by the loss of AtRLP23. Together, our findings provide a solid theoretical foundation and valuable germplasm resources for the development of green, safe, and effective strategies to control Sclerotinia stem rot in *B. napus*.

## 2. Results

### 2.1. BnaSOBIR1 Interacts with Multiple BnaRLP-G13 Members

In our previous study, we showed that BnaSOBIR1 interacted with BnaRLP-G13-1 to activate immune signaling in *B. napus*. To further determine whether other BnaSOBIR1-interacting proteins, BnaSOBIR1-1 and BnaSOBIR1-2, were individually used as bait proteins to screen a *B. napus* membrane-based yeast two-hybrid (MbYTH) library. The screening results were statistically analyzed, and only those LRR-RLPs that appeared in both BnaSOBIR1-1 and BnaSOBIR1-2 screenings and were potentially associated with plant immunity were selected for further analysis.

**Figure 1:**
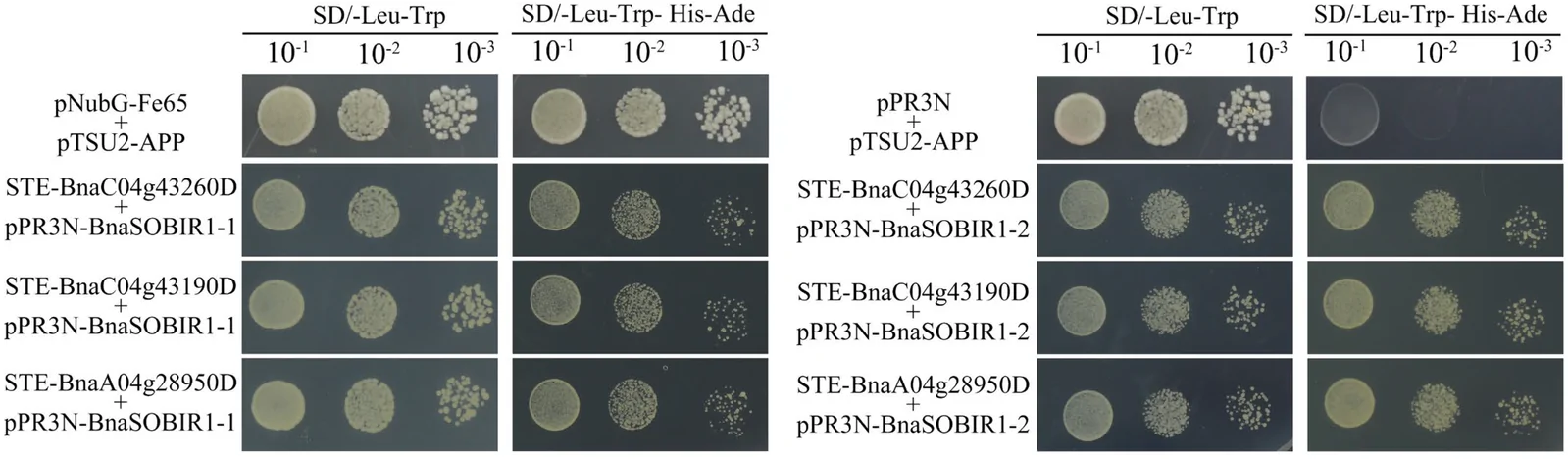
Pairwise Y2H assays between BnaSOBIR1-1/2 and candidate proteins. BnaSOBIR1-1/2 were fused with pPR3-N, three candidate proteins (BnaC04g43260D, BnaC04g43190D, and BnaA04g28950D) were fused with pBT3-STE. Positive control: pNubG-Fe65 + pTSU2-APP. Negative control: pPR3-N + pTSU2-APP. Yeast growth was assessed after 5 days.

Three candidate proteins meeting these criteria were identified (BnaC04g43260D, BnaC04g43190D, and BnaA04g28950D). Pairwise Y2H assays were then conducted to validate their interactions with BnaSOBIR1-1 and BnaSOBIR1-2. Yeast co-transformants harboring each pair of constructs grew normally on SD/-Leu-Trp-His-Ade selective medium (Figure 1), confirming that the interactions occurred between *Bna*SOBIR1 and the three candidate proteins.

**Figure 2:**
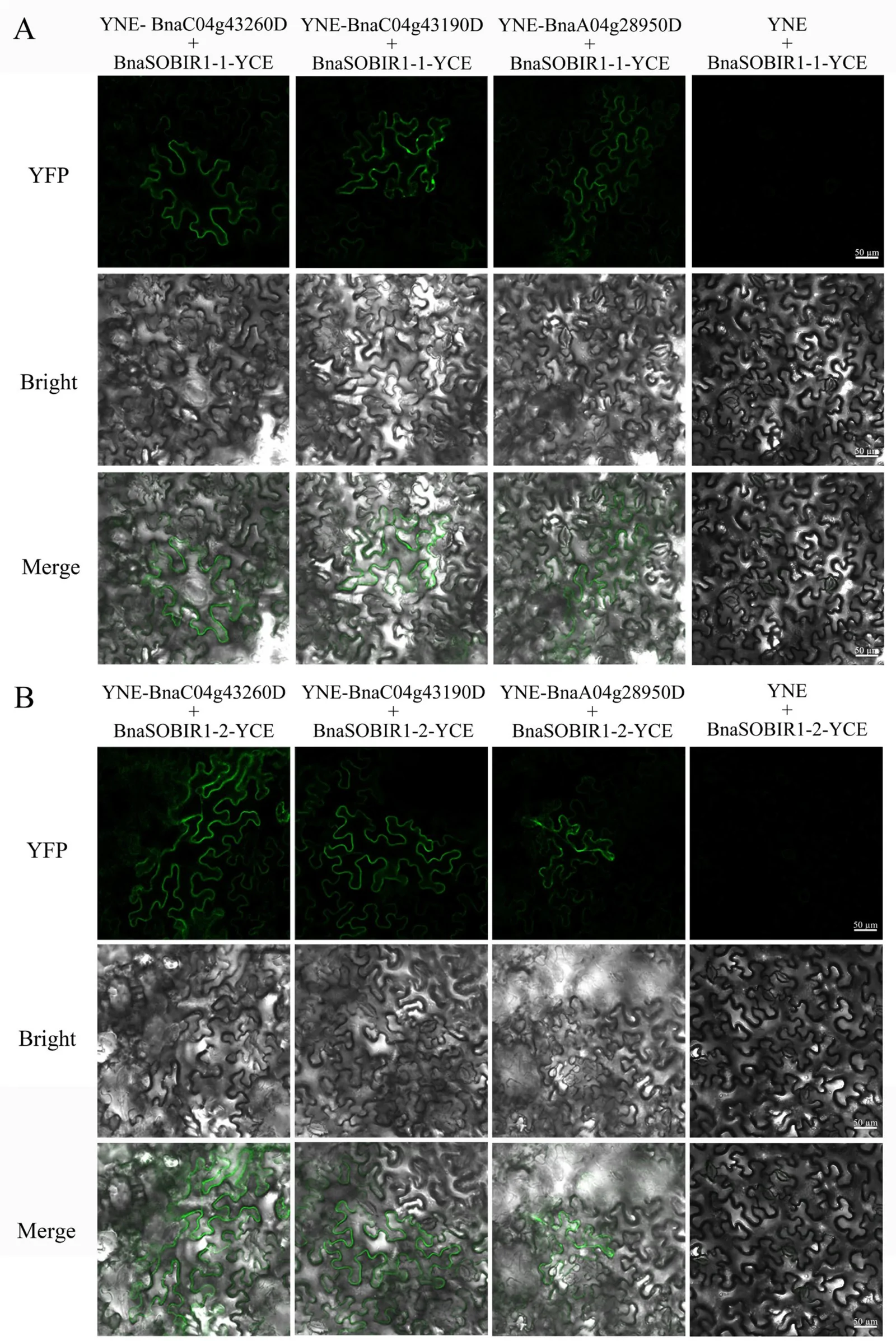
Analysis of interactions between BnaSOBIR1 and candidate proteins by BiFC. (**A**) Interaction analysis between BnaSOBIR1-1 and the candidate proteins. (**B**) Interaction analysis between BnaSOBIR1-2 and the candidate proteins. BnaSOBIR1-1 and BnaSOBIR1-2 were fused to YCE, three candidate proteins (BnaC04g43260D, BnaC04g43190D, and BnaA04g28950D) were fused to YNE. Negative control: YNE + BnaSOBIR1-1/2-YCE. Merged images represent the overlay of YFP fluorescence and bright-field microscopy images. Scale bar = 50 μm.

To further confirm these interactions in planta, bimolecular fluorescence complementation (BiFC) assays were performed. As expected, clear YFP fluorescence signals were observed in the epidermal cells of *Nicotiana benthamiana* leaves co-expressing YNE-fused candidate proteins and YCE-BnaSOBIR1 constructs (Figure 2A,B, Supplementary Figure S1), indicating that BnaSOBIR1-1/2 interacted with all three candidates at the plasma membrane. Taken together, the results from Y2H and BiFC analyses confirmed that, in addition to BnaRLP-G13-1, BnaSOBIR1 interacted with three additional LRR-RLPs in *B. napus* (BnaC04g43260D, BnaC04g43190D, and BnaA04g28950D). Because these three interacting proteins clustered with BnaRLP-G13-1 in the same phylogenetic group (Group 13) [22], they were designated as BnaRLP-G13-2 (BnaC04g43260D), BnaRLP-G13-3 (BnaC04g43190D), and BnaRLP-G13-4 (BnaA04g28950D), respectively.

### 2.2. BnaRLP-G13-1/2/3/4 Share Conserved Motifs and Domain Architectures

To investigate the structural features of the BnaSOBIR1-interacting proteins, we analyzed their gene organization and conserved domains. Gene structure analysis based on resequencing data revealed that *BnaRLP-G13-2/3/4* each consisted of a single exon lacking introns (Supplementary Figures S2–S4). Secondary structure prediction indicated that all three proteins maintained a similar overall conformation and still contained numerous leucine-rich repeat (LRR) motifs (Supplementary Figure S5). In addition, all three proteins had a signal peptide at the N-terminus, while BnaRLP-G13-2 possessed a transmembrane domain located at the C-terminal region (amino acids 843-865). To experimentally determine their subcellular localization, BnaRLP-G13-2/3/4 were fused with eGFP and transiently expressed in *N. benthamiana* leaves. All three proteins showed plasma membrane localization (Supplementary Figure S6). The basic information on BnaRLP-G13 members, including gene IDs, protein characteristics, and genomic locations, is summarized in Supplementary Table S1.

**Figure 3:**
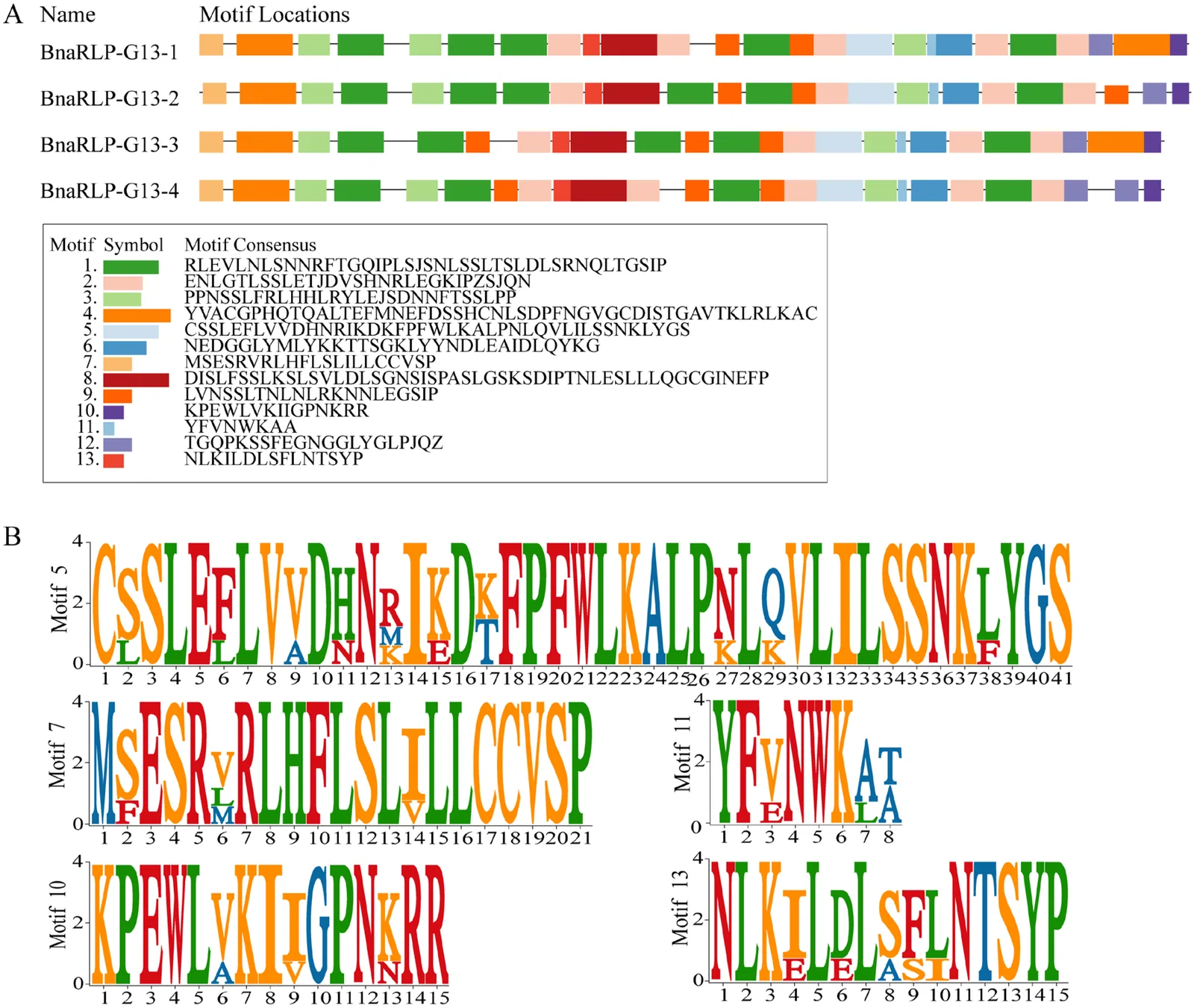
Schematic illustration of the conserved motif organization of BnaRLP-G13-1/2/3/4. (**A**) The positions, numbers, and consensus sequences of the identified motifs within each protein. The scale bars indicate the length of the corresponding proteins. (**B**) Sequence logos of Motifs 5, 7, 10, 11, and 13. The *X*-axis represents the positions of amino acid residues within each motif, and the *Y*-axis represents the information content at each position (in bits).

To further analyze their conserved structural features, motif prediction was performed on these three proteins together with BnaRLP-G13-1, which had been characterized in our previous study. A total of 13 amino acid motifs with different arrangements were identified, with sequence lengths ranging from 8 to 50 residues. The motif composition and positional distribution were predicted using the MEME Suite. Motifs 1 and 2 were the most conserved and were present in all four proteins with relatively high occurrence frequencies (Figure 3A). In contrast, Motifs 5, 7, 10, 11, and 13 appeared only once in each protein but showed high sequence consistency and strong conservation in the corresponding regions. These five motifs were enriched in hydrophobic residues (Figure 3B), including leucine (L), valine (V), isoleucine (I), and phenylalanine (F), suggesting that they might have participated in protein folding, membrane association, or specific protein–protein interactions.

### 2.3. BnaRLP-G13-1/2/3/4 Contain Diverse Stress- and Hormone-Responsive Cis-Elements

**Figure 4:**
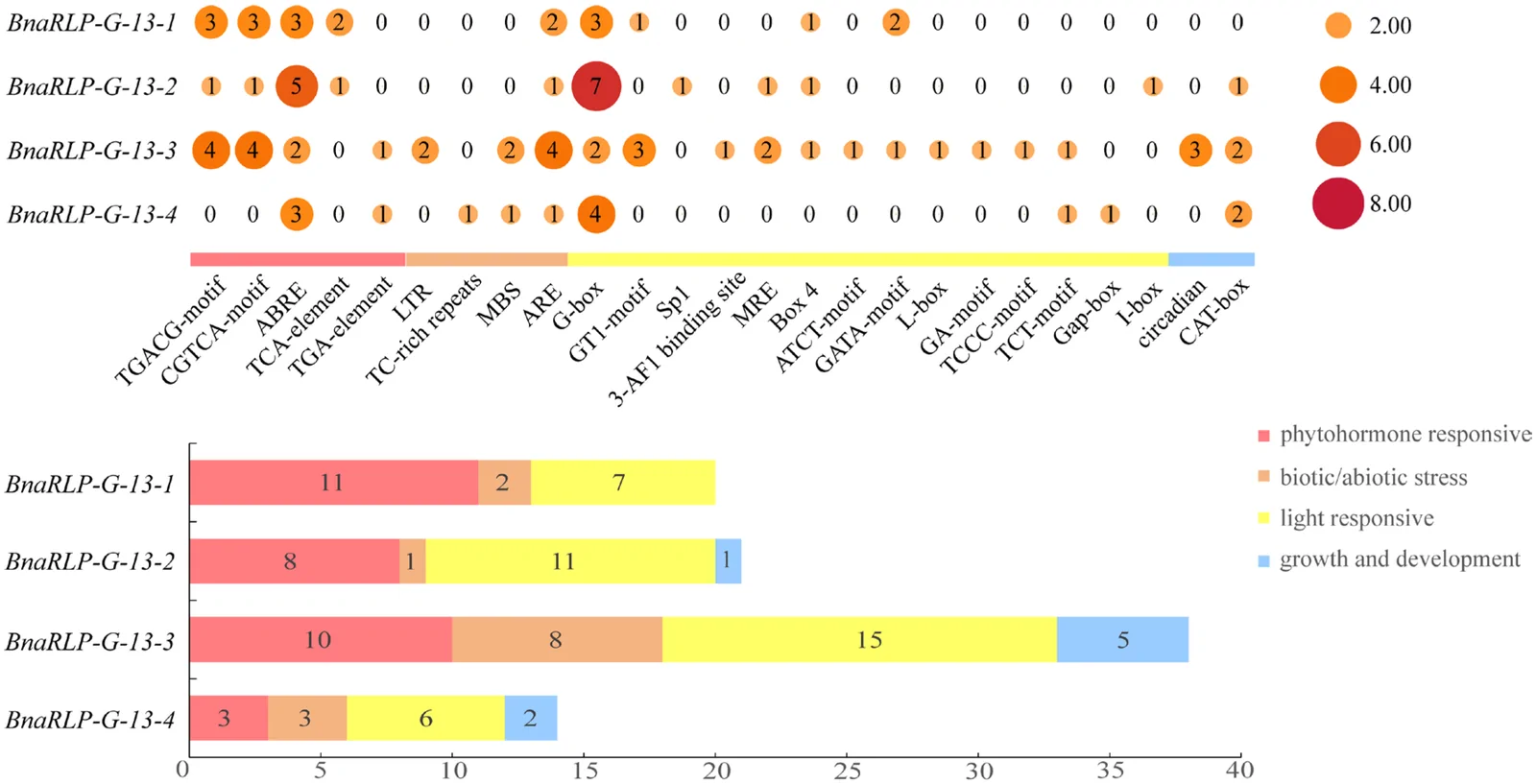
Promoter cis-acting element analysis of *BnaRLP-G13-1/2/3/4* genes. Different categories of cis-acting elements were distinguished by different colors. The numbers, color intensity, and circle sizes represent the abundance of the corresponding cis-acting elements.

Promoter-associated cis-acting elements play essential roles in regulating gene expression. The PlantCARE database was applied to predict cis-regulatory elements within the promoters of *BnaRLP-G13-1/2/3/4*. In total, 25 categories of cis-acting elements were identified among the four promoter regions, included phytohormone responsive elements, biotic/abiotic stress elements, light-responsive elements, as well as growth and development elements (Figure 4).

The promoter of *BnaRLP-G13-4* contained markedly fewer hormone-responsive elements than the other three genes, whereas the promoter of *BnaRLP-G13-1* lacked growth- and development-related elements. Both *BnaRLP-G13-2* and *BnaRLP-G13-3* promoters harbored abundant light-responsive elements; however, those in *BnaRLP-G13-2* were mainly concentrated in G-box elements, while the *BnaRLP-G13-3* promoter contained eleven distinct types of light-responsive elements. Notably, ABRE, ARE, and G-box elements were widely distributed in all four gene promoters (Figure 4), indicating that these genes were closely associated with stress tolerance and growth and development in *B.napus*. In addition, they were extensively regulated by methyl jasmonate (MeJA) and SA signaling pathways, both of which are closely associated with plant immunity.

### 2.4. BnaRLP-G13 Clade Members Exhibit Distinct Transcriptional Responses to S. sclerotiorum

**Figure 5:**
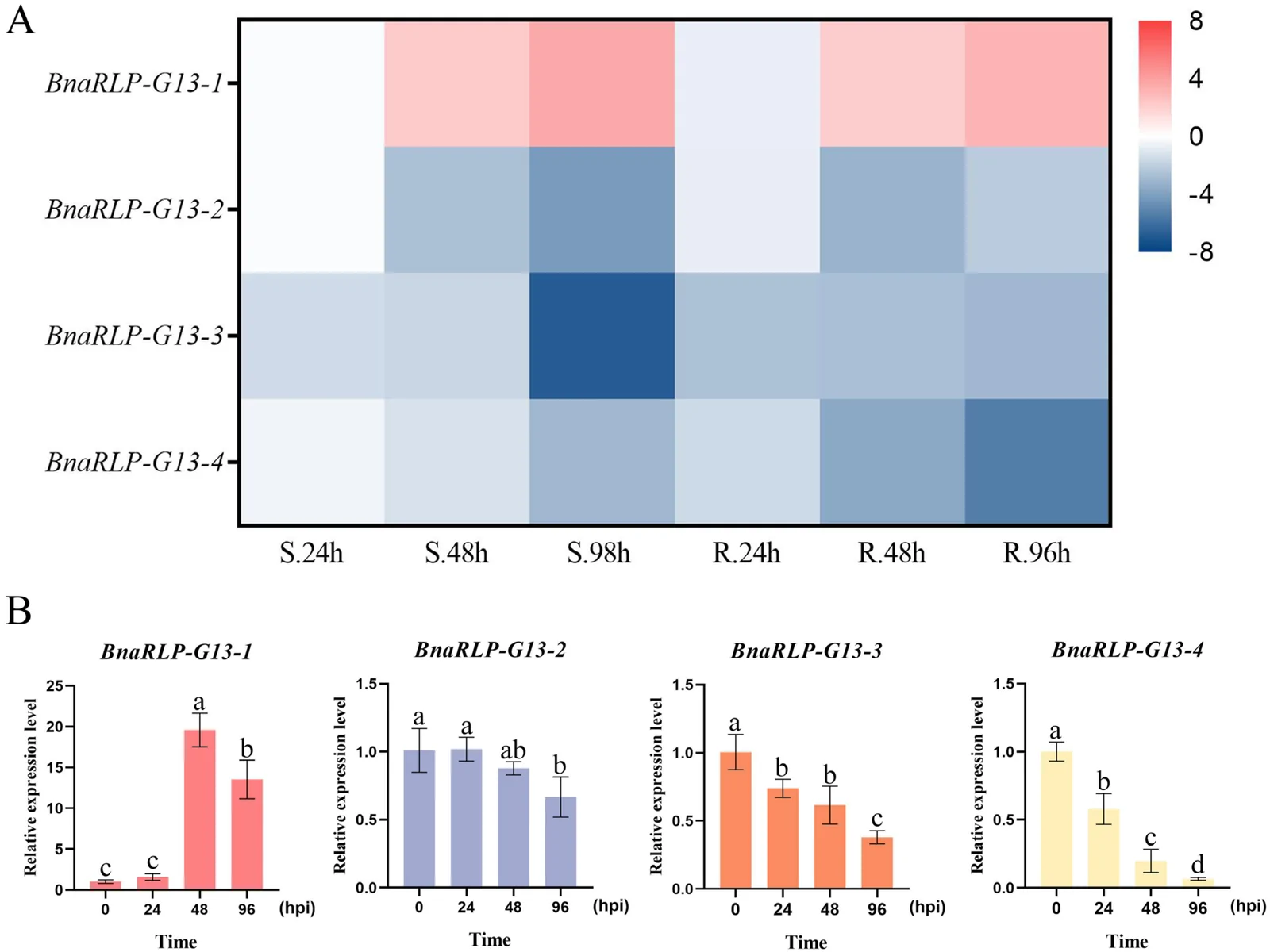
Expression of *BnaRLP-G13-2*/*3*/*4* following *Sclerotinia sclerotiorum* infection. (**A**) Heatmap showing the expression patterns of *BnaRLP-G13-1*/*2*/*3*/*4* in resistant (R) and susceptible (S) *Brassica napus* lines at 24, 48, and 96 h post-inoculation (hpi) with *S. sclerotiorum*. Transcript per million values obtained from the GEO database were used for visualization. (**B**) Relative expression levels of *BnaRLP-G13-1*/*2*/*3*/*4* in XY15 following *S. sclerotiorum* infection at 0, 24, 48, and 96 hpi, as determined by RT-qPCR. Expression levels were normalized to *BnaUBC9*. Different letters indicate statistically significant differences (*p* < 0.05).

To investigate the expression profiles of *BnaRLP-G13-2*/*3*/*4* during disease resistance in *B. napus*, RNA-seq data from *B. napus* leaves at different stages after *S. sclerotiorum* infection were analyzed together with *BnaRLP-G13-1*. The expression patterns of *BnaRLP-G13-1*/*2*/*3*/*4* in the transcriptome data were visualized as heatmaps (Figure 5A). Compared with the early stage of infection, the transcript levels of *BnaRLP-G13-1* increased significantly with prolonged infection time in both types of cultivars. Conversely, the transcript levels of *BnaRLP-G13-2/3/4* decreased markedly as infection progressed.

To validate the reliability of the transcriptome data, reverse transcription quantitative PCR (RT-qPCR) analysis was conducted (Figure 5B). The results showed that, compared with 0 h post-inoculation (hpi), the expression levels of *BnaRLP-G13-3* and *BnaRLP-G13-4* were significantly reduced at 24 hpi. With further progression of infection, the transcript level of *BnaRLP-G13-2* also exhibited a significant decrease at 96 hpi. In contrast, the transcript level of *BnaRLP-G13-1* increased continuously with infection time. Taken together, the results were largely consistent with the RNA-seq data and further confirmed the distinct transcriptional responses of *BnaRLP-G13* members during *S. sclerotiorum* infection.

### 2.5. BnaRLP-G13-2/3/4 Enhance Plant Resistance to S. sclerotiorum

**Figure 6:**
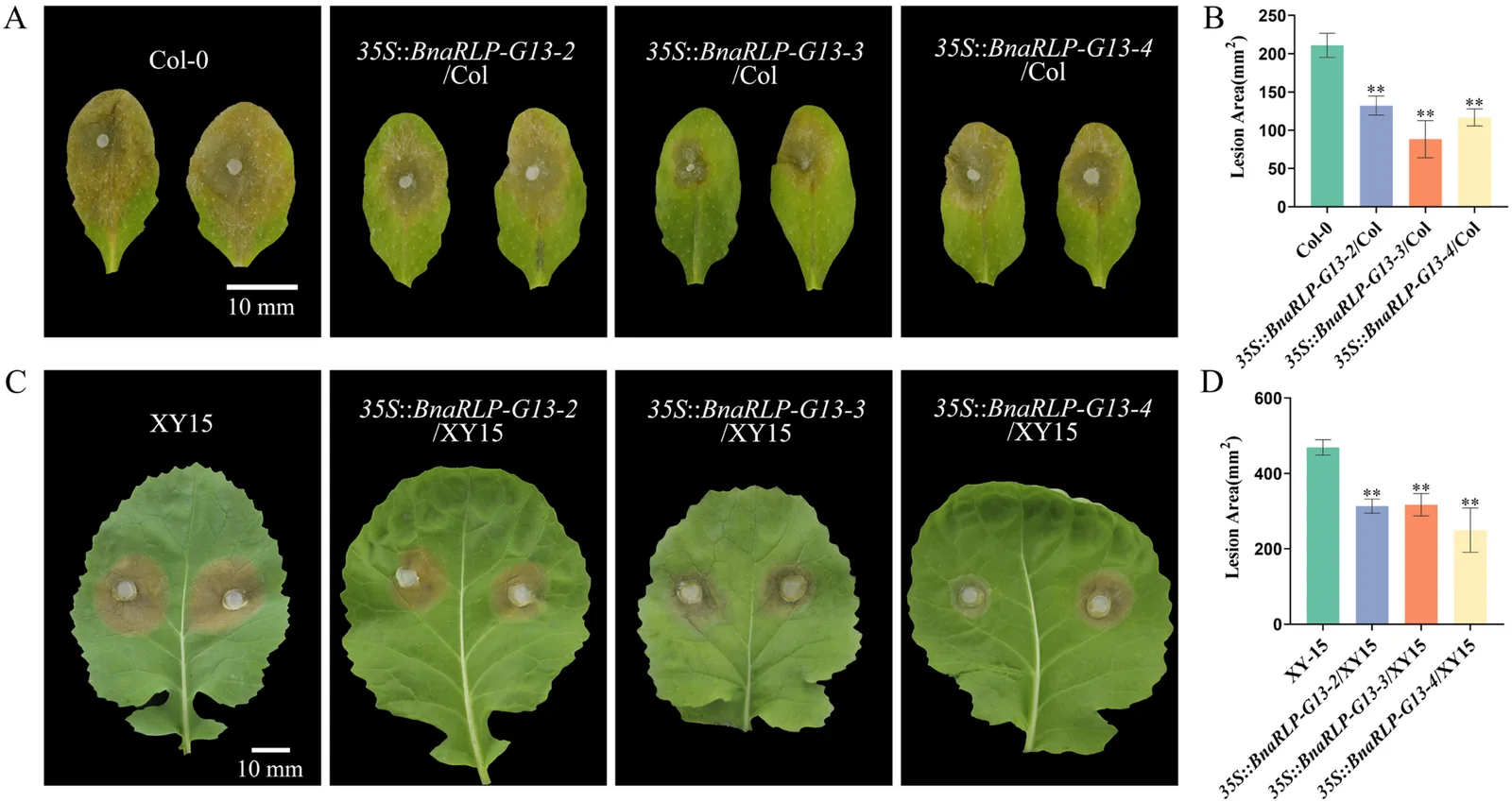
Resistance of *BnaRLP-G13-2/3/4*-overexpressing plants to *S. sclerotiorum*. (**A**,**B**) Disease phenotypes of detached leaves from Col-0 and *Arabidopsis thaliana* plants overexpressing *BnaRLP-G13-2/3/4* following infection by wild-type *S. sclerotiorum*. The plants were inoculated with 2-mm-diameter mycelial plugs. Images were captured at 48 hpi. Scale bars = 10 mm. Lesion areas were quantified using ImageJ (version 1.0). (**C**,**D**) Disease phenotypes of detached leaves from XY15 and *B. napus* plants overexpressing *BnaRLP-G13-2/3/4* following inoculation with wild-type *S. sclerotiorum*. Two mycelial plugs were placed on each leaf. The plants were inoculated with 5-mm-diameter mycelial plugs. Images were captured at 48 hpi. Scale bars = 10 mm. Lesion areas were quantified using ImageJ. For each genotype, three leaves were used for each treatment, with three independent biological experiments performed. Statistical significance was determined using one-way ANOVA followed by Dunnett’s test (** p < 0.01). Error bars represent SD.

With the aim of further clarifying the roles of *BnaRLP-G13-2*/*3*/*4* during the interaction between *B. napus* and *S. sclerotiorum*, these genes were individually overexpressed in both *A. thaliana* and *B. napus*. Overexpression of *BnaRLP-G13-2*/*3*/*4* did not affect plant growth phenotypes in either *A. thaliana* or *B. napus* (Supplementary Figures S7 and S8). Following *S. sclerotiorum* infection, the lesion areas of 35S-*BnaRLP-G13-2*/*3*/*4*/Col overexpression plants were markedly smaller than those of the Col-0 control, with lesion sizes reduced by approximately 30% (Figure 6A,B).

In *B. napus*, transgenic lines with the highest expression levels of *BnaRLP-G13-2*/*3*/*4* were selected for disease resistance assays (Supplementary Figure S8). Consistently, the lesion areas of *BnaRLP-G13-2*/*3*/*4* overexpression plants were significantly smaller than those of the XY15 control, showing an approximate 50% reduction in infected area (Figure 6C,D). Collectively, overexpression of *BnaRLP-G13-2/3/4* was found to enhance resistance to *S. sclerotiorum* in plants, as revealed by these results.

### 2.6. BnaRLP-G13-2/3/4 Exhibit AtRLP23-like Immune Functions

**Figure 7:**
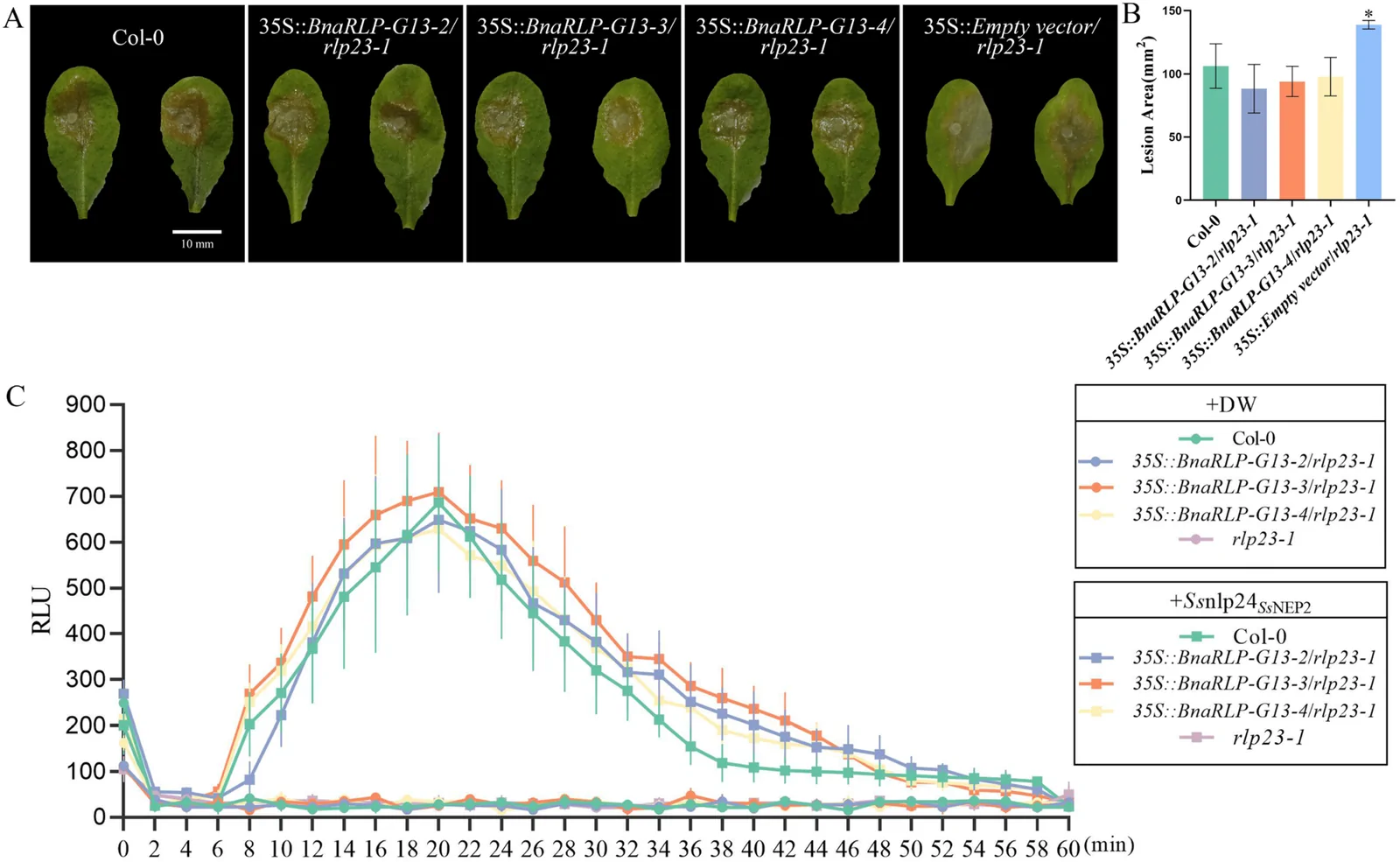
*BnaRLP-G13-2/3/4* restore *AtRLP23*-mediated immune responses. (**A**,**B**) Lesion areas on leaves of Col-0, the complementation lines and *35S::Empty vector*/*rlp23-1* following *S. sclerotiorum* infection. The plants were inoculated with 2-mm-diameter mycelial plugs. Scale bar = 10 mm. Lesion area was recorded at 36 hpi. Lesion areas were quantified using ImageJ. For each genotype, three leaves were used for each treatment, with three independent biological experiments performed. Statistical significance was determined using one-way ANOVA followed by Dunnett’s test (* p < 0.05). Error bars represent SD. (**C**) ROS burst was measured in leaf discs from five-week-old *A. thaliana* plants (Col-0, *rlp23-1*, and the complementation lines) after treatment with 1 μM *Ss*nlp24*_Ss_*_NEP2_, or H_2_O. ROS levels were expressed as relative light units (RLUs). Values represent means ± SD of three independent biological replicates, with 9 leaves analyzed in each replicate.

Phylogenetic analysis showed that BnaRLP-G13-2/3/4 clustered together with AtRLP23 in the same clade, indicating a high degree of sequence homology between them [23]. This suggested that *BnaRLP-G13-2*/*3*/*4* might possess functions similar to those of *AtRLP23*, which required further experimental validation. Infection assays demonstrated that loss of *AtRLP23* function increased susceptibility to *S. sclerotiorum*. When *BnaRLP-G13-2*/*3*/*4* were individually introduced into the *rlp23-1* mutant background (Supplementary Figure S9), the lesion areas after infection were reduced to levels comparable to those of the wild-type control (Figure 7A,B), indicating that the immune defects caused by *AtRLP23* mutation were effectively restored.

Reactive oxygen species (ROS) play crucial roles in enhancing plant defense and stress tolerance by sensing and integrating environmental stress signals and activating downstream defense networks. A 24-amino acid peptide (*Ss*nlp24*_Ss_*_NEP2_) derived from *S. sclerotiorum* was previously reported to play an important role in triggering ROS production in plants. To further investigate the roles of *BnaRLP-G13-2*/*3*/*4* in resistance against *S. sclerotiorum*, ROS burst assays were performed. The results showed that treatment with *Ss*nlp24*_Ss_*_NEP2_ failed to induce ROS production in the *rlp23-1* mutant, whereas a clear ROS burst was restored in the complementation lines expressing *BnaRLP-G13-2*/*3*/*4* (Figure 7C). The induced fluorescence intensity was comparable to that observed in Col-0 treated with the peptide.

### 2.7. BnaRLP-G13-2/3/4 Activate Plant Immunity Through Ssnlp24_SsNEP2_

**Figure 8:**
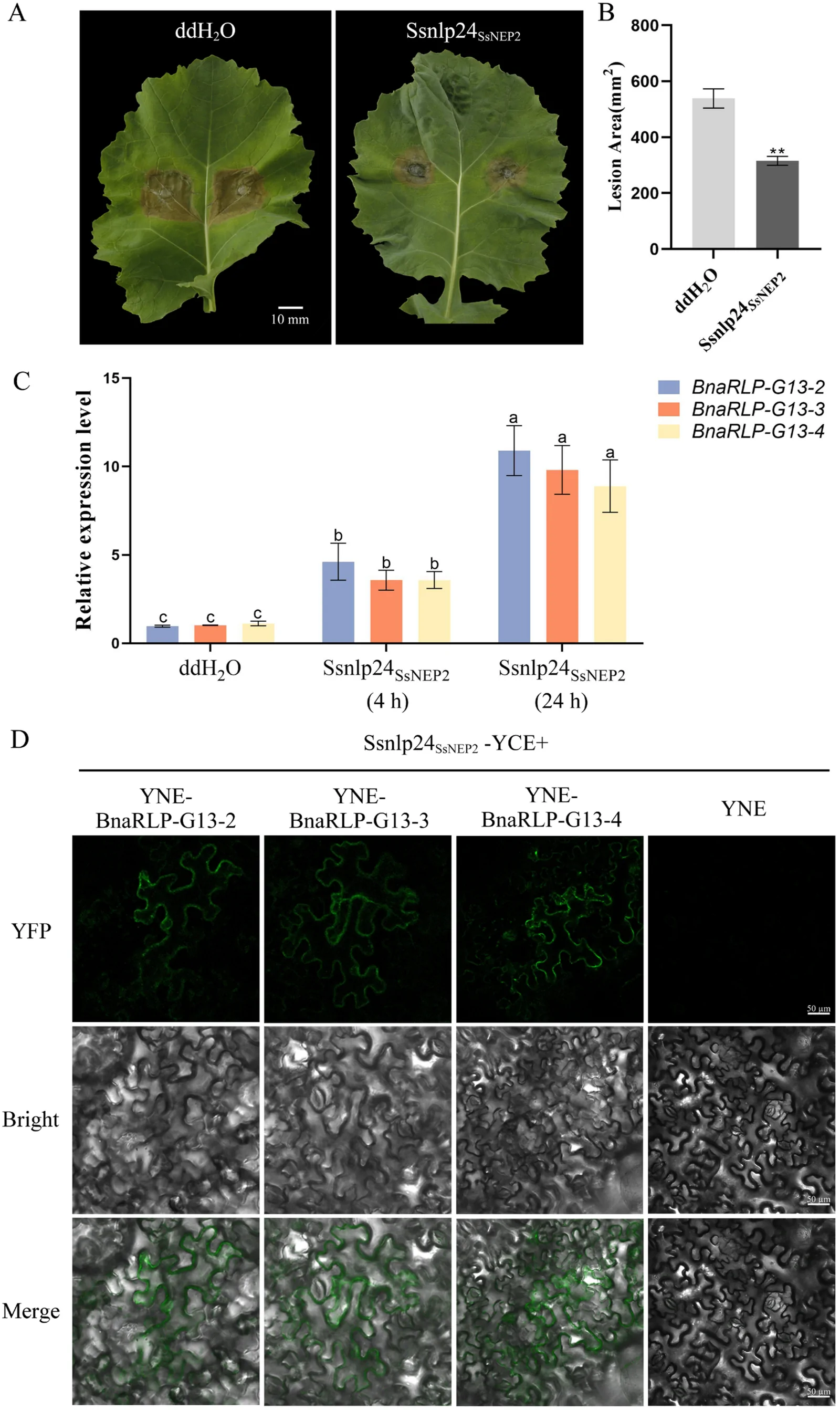
BnaRLP-G13-2/3/4 mediate immune responses to Ssnlp24*_Ss_*_NEP2_. (**A**,**B**) XY15 were treated with ddH_2_O or 1 μM *Ss*nlp24*_Ss_*_NEP2_ for 24 h before inoculation with *S. sclerotiorum*. Two mycelial plugs (5-mm-diameter) were placed on each leaf. Images were measured at 48 hpi and quantified using ImageJ. Scale bar = 10 mm. For each genotype, three leaves were used for each treatment, with three independent biological experiments performed. Statistical significance between ddH_2_O and 1 μM Ssnlp24*_Ss_*_NEP2_ treatment was analyzed using Student’s *t*-test (** *p* < 0.01). Error bars represent SD. (**C**) Relative expression levels of *BnaRLP-G13-2/3/4* in XY15 following treatment with 1 μM Ssnlp24_SsNEP2_ at 4 and 24 h, determined by RT-qPCR. Different letters indicate statistically significant differences (*p* < 0.05). (**D**) The association between BnaRLP-G13-2/3/4 and Ssnlp24*_Ss_*_NEP2_ was examined by BiFC. *Bna*RLP-G13-2/3/4 were fused to YNE and Ssnlp24*_Ss_*_NEP2_ to YCE. YFP fluorescence was observed, and merged images combined YFP and bright-field signals. Scale bar = 50 μm.

Previous work demonstrated that external application of Ssnlp24_SsNEP2_ can confer enhanced resistance against *S. sclerotiorum* and oomycete pathogens in *A. thaliana* [22,24]. Consistent with these findings, Ssnlp24_SsNEP2_ treatment also significantly increased resistance to *S. sclerotiorum* in *B. napus* (Figure 8A,B). To investigate whether BnaRLP-G13 members respond to Ssnlp24_SsNEP2_, the transcript levels of BnaRLP-G13-2/3/4 were analyzed after peptide treatment. RT-qPCR analysis revealed that the transcript of *BnaRLP-G13-2/3/4* was significantly induced by Ssnlp24_SsNEP2_ treatment (Figure 8C), indicating that these genes are responsive to the conserved fungal peptide. To determine whether BnaRLP-G13-2/3/4 are involved in Ssnlp24_SsNEP2_ perception, BiFC assays were conducted. As shown in Figure 8D, YFP fluorescence signals were detected in leaf epidermal cells co-infiltrated with Ssnlp24_SsNEP2_-YCE and YNE-BnaRLP-G13-2/3/4, supporting the association between Ssnlp24_SsNEP2_ and BnaRLP-G13-2/3/4. Moreover, the interaction signals were localized to the plasma membrane.

**Figure 9:**
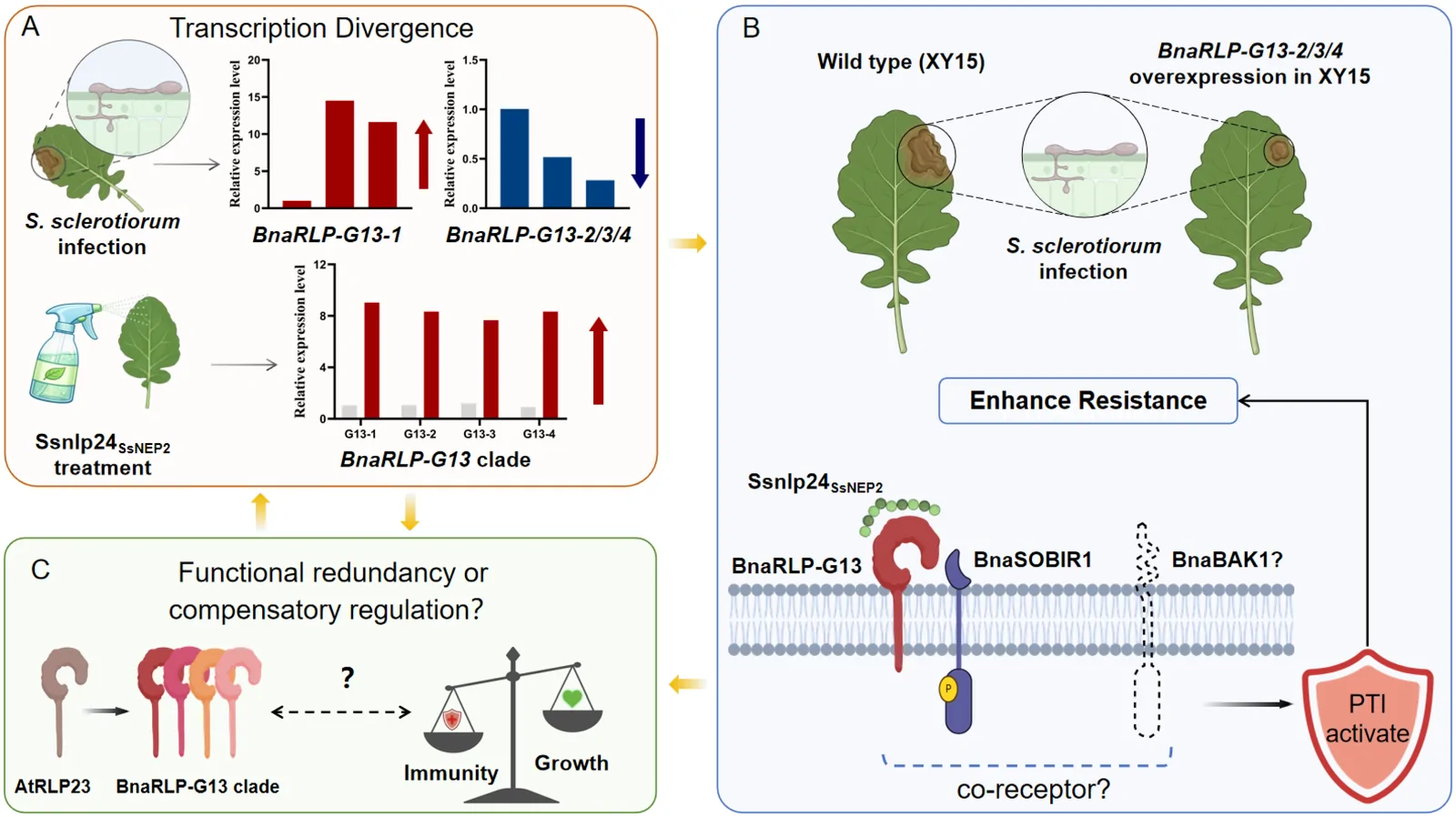
A model of BnaSOBIR1-associated BnaRLP-G13-mediated immunity against *S. sclerotiorum* in *B. napus*. (**A**) BnaRLP-G13 members exhibit distinct transcriptional responses under different immune stimuli. During *S. sclerotiorum* infection, *BnaRLP-G13-1* is induced, whereas *BnaRLP-G13-2/3/4* are downregulated. In contrast, treatment with the conserved peptide Ssnlp24_SsNEP2_ induces the expression of all *BnaRLP-G13* members. (**B**) BnaRLP-G13 receptors are involved in Ssnlp24_SsNEP2_ perception and associate with BnaSOBIR1 to activate PTI, thereby enhancing resistance to *S. sclerotiorum*. A putative co-receptor is indicated by dashed outlines because its involvement has not been experimentally validated in this study. (**C**) The expansion of the BnaRLP-G13 family in *Brassica napus* suggests potential functional redundancy or compensatory regulation while maintaining conserved immune functions. The molecular basis underlying these regulatory mechanisms remains to be investigated.

Collectively, these results support a model whereby multiple BnaSOBIR1-associated BnaRLP-G13 receptors contribute to resistance against *S. sclerotiorum*. *BnaRLP-G13* members showed diverse transcriptional responses during *S. sclerotiorum* infection, whereas all members were induced by Ssnlp24_SsNEP2_ treatment, suggesting distinct regulatory responses between pathogen infection and single elicitor perception. In response to Ssnlp24_SsNEP2_, BnaRLP-G13 receptors associate with BnaSOBIR1 and activate PTI signaling, thereby enhancing resistance to *S. sclerotiorum*. Given the expansion of the BnaRLP-G13 family in *B. napus*, these receptors may function through redundancy or compensatory regulation while maintaining conserved immune functions (Figure 9).

## 3. Discussion and Conclusions

Previous studies have demonstrated that SOBIR1 functions as a common co-receptor for multiple RLPs and plays a crucial role in plant innate immunity. Our previous work showed that BnaSOBIR1 interacts with BnaRLP-G13-1 and contributes to resistance against *S. sclerotiorum* in *B. napus* [22]. Nevertheless, a comprehensive characterization of the interaction partners of BnaSOBIR1 in *B. napus* is still lacking. We previously identified six BnaSOBIR1 family members, which showed high conservation, particularly within their kinase domains, and were classified into two groups based on phylogenetic analysis. BnaSOBIR1-1 (BnaA03g14760D) and BnaSOBIR1-2 (BnaA04g18590D) were selected as representatives of the two groups and independently used as bait proteins for screening a *B. napus* membrane-based yeast library. The candidate interactors identified using the two BnaSOBIR1 homologs as baits were compared to improve the confidence of interaction identification.

Ultimately, three immune-related LRR-RLPs (BnaC04g43260D, BnaC04g43190D, and BnaA04g28950D) were consistently identified in both screening systems, and their stable interactions with both BnaSOBIR1 homologs were further validated. This selection was based on their classification within the G13 RLP clade, their close relationship with the previously characterized BnaRLP-G13-1 and AtRLP23, and their conserved LRR-RLP domain organization [25]. Since BnaRLP-G13-1 has been previously shown to interact with BnaSOBIR1 and contribute to resistance against *S. sclerotiorum* [22], characterization of additional G13 members allowed us to investigate whether similar immune functions are conserved among duplicated RLP receptors in *B. napus*. Although additional RLP or RLK candidates were detected that interacted with only one of the two BnaSOBIR1 proteins, the present study focused on the conserved immune receptors that were shared by both interaction screens. SOBIR1 functions as a conserved co-receptor for numerous immune-related RLPs and is required for receptor complex activation during plant immunity [6,26]. Consistent with this role, the identification of multiple BnaSOBIR1-associated receptors suggests that BnaSOBIR1 may participate in the coordination of immune signaling in *B. napus*.

Previous studies have shown that RLP-SOBIR1 receptor complexes generally require additional SERK family co-receptors for full immune signaling. In Arabidopsis, AtRLP23 forms an active receptor complex involving SOBIR1 and the co-receptor BAK1 after ligand perception to initiate downstream immune responses. The interaction between BnaRLP-G13 proteins and BnaSOBIR1 was supported by both MbY2H and BiFC validation assays (Figure 1 and Figure 2, Supplementary Figure S1). Future studies using endogenous interaction analyses will help further clarify the physiological significance and regulatory mechanisms underlying this receptor association. Whether BnaRLP-G13 activates downstream signaling through a similar receptor complex remains unclear and requires further investigation.

Resequencing analysis revealed that *BnaRLP-G13-2/3/4* are each composed of a single exon without introns (Supplementary Figures S2–S4). This gene structure shows slight differences from the annotations in public databases, which may be attributable to cultivar-specific variation. Notably, BnaRLP-G13-1 and AtRLP23 are also intronless, raising the possibility that intron loss or genome structural simplification may have contributed to the evolutionary diversification of this RLP clade. Although computational prediction identified a transmembrane domain only in BnaRLP-G13-2, subcellular localization analysis showed that BnaRLP-G13-2/3/4 were all localized to the plasma membrane. This discrepancy may be explained by the limitations of computational prediction methods in identifying divergent or non-canonical membrane-targeting features.

Motif analysis identified a total of 13 amino acid motifs with distinct arrangements. Among them, motifs 1 and 2 were highly conserved across BnaRLP-G13-1/2/3/4 (Figure 3A), suggesting that they may be associated with maintaining overall protein structural stability or core immune functions. In contrast, motifs 5, 7, 10, 11, and 13 occurred only once in each protein but exhibited high amino acid composition consistency and were enriched in hydrophobic residues (Figure 3B). These motifs may contribute to protein folding, membrane-associated structural formation, or specific ligand recognition, thereby allowing a certain degree of functional diversification while preserving conserved core functions.

Promoter analysis revealed that BnaRLP-G13 members are broadly enriched in cis-elements related to hormone signaling, stress responses, and light responsiveness (Figure 4). Beyond their roles in plant defense, RLPs have also been shown to participate in growth and developmental regulation [27]. Among the four *BnaRLP-G13* members, *BnaRLP-G13-1* contained the highest proportion of hormone-responsive cis-elements, particularly those associated with SA and JA signaling. Both SA and JA pathways serve as key regulators of plant resistance to pathogen attack and are closely associated with the regulation of immune responses to *S. sclerotiorum* [28,29]. The enrichment of SA- and JA-responsive cis-elements in the *BnaRLP-G13-1* promoter may therefore facilitate its transcriptional activation during pathogen infection. Notably, *BnaRLP-G13-3* harbored a markedly higher abundance and diversity of light-responsive elements in its promoter. This suggests that *BnaRLP-G13-3* may integrate light signaling with immune regulation, potentially contributing to the coordination between defense and growth. In contrast, the promoter of *BnaRLP-G13-1* lacked obvious growth- and development-related cis-elements, which may indicate a relatively stronger association with immune regulation than with broader growth-related processes.

Transcriptomic and RT-qPCR analyses showed that *BnaRLP-G13-2/3/4* transcripts were downregulated during *S. sclerotiorum* infection, whereas *BnaRLP-G13-1* was induced (Figure 5). In contrast, treatment with the conserved peptide Ssnlp24_SsNEP2_ significantly enhanced the transcript levels of all *BnaRLP-G13* members (Figure 8C, Supplementary Figure S10), indicating that their transcriptional responses are dependent on the nature of the stimulus. This difference may result from the distinct regulatory responses induced by pathogen infection and purified immune elicitors. Ssnlp24_SsNEP2_ is a defined pathogen-derived signal that primarily activates early pattern-triggered immunity. In contrast, *S. sclerotiorum* infection involves more complex regulatory networks. These processes may include pathogen virulence factors, phytohormone signaling, and immune feedback regulation [30,31]. Unlike treatment with a single conserved peptide, infection by living *S. sclerotiorum* involves complex interactions between pathogen-derived signals and host regulatory networks. *S. sclerotiorum* produces multiple virulence factors that can modulate host immune responses during infection. For example, the effector SsPINE1 has been shown to suppress plant immunity and promote fungal virulence [32].In addition, other fungal effectors can interfere with plant defense pathways by targeting immune-related components, such as chloroplast-associated calcium signaling proteins and hypersensitive response-related proteins [32,33,34]. Therefore, the reduced expression of *BnaRLP-G13-2/3/4* during pathogen infection does not necessarily indicate diminished immune function but may reflect the dynamic regulation of immune receptor expression during the plant-pathogen interaction.

Consistent with this interpretation, overexpression (Figure 6), genetic complementation (Figure 7), and receptor interaction assays (Figure 8) demonstrated that *BnaRLP-G13-2/3/4* function as positive regulators of resistance to *S. sclerotiorum*. Earlier studies have revealed that the activity of cell-surface immune receptors can be regulated at multiple levels beyond transcription, including receptor complex assembly, protein turnover, and post-translational modification [35,36,37]. However, these regulatory mechanisms have not been examined in the present study. Further studies are needed to determine whether BnaRLP-G13 proteins are regulated at the post-transcriptional or post-translational level during pathogen infection. Unlike the single *AtRLP23* gene in Arabidopsis, *B. napus* contains multiple closely related *BnaRLP-G13* members. We speculate that these receptors may have undergone regulatory divergence while retaining conserved immune functions. Whether this phenomenon reflects functional redundancy, coordinated regulation among *BnaRLP-G13* members, or other regulatory mechanisms requires further investigation.

In addition, the downstream signaling mechanism mediated by BnaRLP-G13 remains unclear. In other plant species, RLP-SOBIR1 receptor complexes have been shown to recruit the co-receptor BAK1 to initiate downstream immune signaling [10,12,38,39]. Previous research has demonstrated that other SERK family members act as co-receptors in different RLP-mediated immune pathways [26]. However, whether BnaRLP-G13 associates with BAK1 or other SERK family members to form a similar receptor complex remains to be determined.

Taken together, our results suggest that multiple BnaRLP-G13 members function as BnaSOBIR1-associated immune receptors in rapeseed. *BnaRLP-G13* members showed different transcriptional responses to *S. sclerotiorum* infection and Ssnlp24_SsNEP2_ treatment. However, they shared conserved immune functions. The Arabidopsis system supported the conserved immune function of BnaRLP-G13 members through complementation of the *AtRLP23* pathway, whereas the *B. napus* overexpression system provided evidence for their potential roles in enhancing resistance against *S. sclerotiorum* in the native crop background. All members involved in Ssnlp24_SsNEP2_ perception interacted with BnaSOBIR1 and enhanced resistance to *S. sclerotiorum*. These findings suggest that transcriptional divergence is not necessarily accompanied by functional divergence. The increased number of *BnaRLP-G13* members in *B. napus* suggests potential functional redundancy or coordinated regulation. The underlying mechanism remains to be determined (Figure 9).

As an allotetraploid crop derived from the hybridization of *B. rapa* and *B. oleracea*, *B. napus* retains duplicated gene copies that may contribute to the diversification of immune responses [40]. Duplicated RLP genes retained from the two progenitor genomes may provide functional redundancy and/or diversification, thereby enhancing the robustness and adaptability of immune recognition. The maintenance of multiple *BnaRLP-G13* members may allow plants to sustain immune capacity under changing pathogen pressures by providing complementary receptor activities or increasing the flexibility of pathogen perception. Similar expansion and diversification of plant immune receptor families have been reported to contribute to adaptation against diverse pathogens [6,41]. The conserved immune activity of *BnaRLP-G13-1/2/3/4* suggests that these receptors may serve as candidate targets for resistance improvement. From a breeding perspective, *BnaRLP-G13* members represent potential resources for improving resistance to *S. sclerotiorum*. Recent studies have highlighted the potential of RLP-based immunity for crop improvement through receptor transfer or engineering [20]. Therefore, combining different *BnaRLP-G13* members or favorable alleles through breeding, or optimizing receptor activity through genome editing targeting regulatory regions or key functional domains (such as C-terminal region), may provide new strategies for enhancing resistance in *B. napus*. However, these approaches require further genetic and field validation.

Despite these advances, several limitations remain in the present study. First, the functions of *BnaRLP-G13* members still require validation using loss-of-function mutants. However, *B. napus* is an allotetraploid species with multiple homologous gene copies. Its long life cycle also makes mutant generation time-consuming. Furthermore, the high sequence similarity among BnaRLP-G13 members makes it challenging to achieve gene-specific suppression using approaches such as VIGS or RNA interference without potential effects on closely related homologs. Therefore, reliable gene-specific loss-of-function materials for individual BnaRLP-G13 members are currently unavailable. Second, the molecular basis of Ssnlp24_SsNEP2_ recognition remains unclear. Direct biochemical evidence demonstrating the interaction between BnaRLP-G13 members and Ssnlp24_SsNEP2_ is still lacking and requires further validation. The structural mechanisms underlying receptor complex assembly also require further investigation. This includes the interaction interfaces between BnaRLP-G13 members and their associated co-receptors. In addition, whether BnaSOBIR1 regulates the protein stability or post-translational modification of BnaRLP-G13 members during immune activation remains unknown. Further studies using appropriate genetic materials and in planta detection approaches will be required to elucidate the regulatory mechanisms of Ssnlp24 _SsNEP2_-BnaRLP-G13-BnaSOBIR1 receptor complexes. Third, the mechanism responsible for the distinct transcriptional responses of *BnaRLP-G13* members during pathogen infection remains unknown. Whether these differences result from functional redundancy, coordinated regulation, or other mechanisms will be investigated in future studies.

## 4. Materials and Methods

### 4.1. MbY2H Library Screening and Pairwise Interaction Assays

A membrane-based Y2H screening was performed using the DUAL membrane system (Dual Systems Biotech, Zurich, Switzerland). The complete coding regions of BnaSOBIR1-1/2 were inserted into the pBT3-STE bait through the SfiI cloning site to generate C-terminal Cub fusion constructs. The bait constructs were transformed into the NMY51 yeast strain for subsequent interaction assays. Bait strains were tested for autoactivation and toxicity on selective media. The *B. napus* (XY15) membrane yeast cDNA library constructed in the pPR3-N vector was transformed into yeast cells harboring the BnaSOBIR1-1/2 bait constructs for library screening. Yeast transformants were screened on SD/-Leu/-Trp/-His medium supplemented with 5 mM 3AT to select for positive interactions. Colonies that grew on selective media were further restreaked and subjected to plasmid rescue and sequencing to identify candidate interacting proteins. The genomic sequences and annotation information of candidate genes were retrieved from the Ensembl Plants database (http://plants.ensembl.org/index.html, accessed on 1 February 2023).

For interaction validation, full-length cDNA sequences of candidate genes and BnaSOBIR1-1/2 were cloned into the SfiI-linearized pBT3-STE and pPR3-N vectors, respectively, to generate prey and bait constructs. The resulting prey plasmids were transformed into the NMY51 yeast strain already expressing the corresponding bait constructs. Yeast transformants were evaluated for growth on SD/-Leu/-Trp/-His/-Ade selective medium. The primers were listed in Supplementary Table S2.

### 4.2. BiFC Assay

Full-length cDNAs of Ssnlp24*_Ss_*_NEP2_, *BnaSOBIR1-1/2*, and *BnaRLP-G13-2/3/4* were PCR-amplified from cDNA templates using specific primers (Supplementary Table S2). The pSPYNE and pSPYCE vectors encoded the N-terminal and C-terminal portions of yellow fluorescent protein (YFP). Ssnlp24_SsNEP2_ and *BnaSOBIR1-1/2* fragments were cloned into the pSPYCE vector, while *BnaRLP-G13-2/3/4* fragments were inserted into the pSPYNE vector, both using the KpnI and BamHI restriction enzyme recognition sites.

The generated BiFC-resulting constructs were introduced into *Agrobacterium tumefaciens* GV3101 cells. Bacterial suspensions containing the recombinant plasmids were prepared in infiltration buffer consisting of 10 mM MgCl_2_, 10 mM MES, and 150 μM acetosyringone (pH 5.6). Equal amounts of paired bacterial suspensions were combined and infiltrated into *N. benthamiana* leaves. After 36–72 h of incubation, YFP fluorescence signals were examined using an Axio Imager 2 (ZEISS, Oberkochen, Germany). The YFP signal was excited at 470–490 nm and collected within the emission range of 500–540 nm.

### 4.3. Gene Structure, Conserved Motif, and Cis-Regulatory Element Analyses

The exon-intron structures of *BnaRLP-G13* genes were characterized using the Gene Structure Display Server 2.0 [42]. Conserved protein motifs of BnaRLP-G13 members were predicted using the MEME Suite. The analysis was performed with the maximum number of motifs set to 15 and motif lengths restricted to 6–50 amino acids, while the remaining parameters were maintained at default values. Signal peptides (SPs) and transmembrane (TM) regions were predicted using a combination of bioinformatics tools, including SignalP 5.0, SMART, TMHMM Server v2.0, and the Pfam database.

To analyze the promoter regions, genomic regions spanning 2000 bp upstream of the ATG initiation codon (ATG) of each *BnaRLP-G13* gene were extracted from the reference genome of *B*. *napus*. Cis-regulatory elements were identified using the PlantCARE database under default settings [43]. The identified cis-elements were manually classified according to their annotated biological functions, and hormone-, stress-, growth- and development-, and light-responsive elements were retained for comparative analysis. All results were visualized using TBtools (version 2.39) [44]. The websites used in this study are listed in Supplementary Table S3.

### 4.4. Cloning of Target Genes and Vector Generation

The complete coding sequences (CDSs) of *BnaRLP-G13-2/3/4* were amplified from *B. napus* cDNA. For overexpression, the binary vector pCAMBIA1305-3FLAG was cleaved with KpnI and BamHI, and the target CDSs were inserted into the vector by homologous recombination. Gene expression was controlled by the constitutive Cauliflower mosaic virus 35S promoter and terminated by the nopaline synthase terminator. The vector carried the hygromycin gene as the plant selection marker.

Recombinant plasmids were transformed into GV3101 and used for transformation of *A. thaliana* and *B. napus*. The same constructs were also introduced into the *rlp23-1* mutant for complementation assays. The primers used are listed in Supplementary Table S2.

### 4.5. Plant Materials and Fungal Strains

Plants of *A. thaliana* and *B. napus* were grown under controlled conditions at 22 °C with a long-day regime consisting of 16 h illumination and 8 h darkness. The *B. napus* cultivar Xiangyou 15 (XY15), a high-quality rapeseed variety extensively cultivated in the middle and lower regions of the Yangtze River in China, was used in this study. XY15 exhibits moderate resistance to *S. sclerotiorum* and has been routinely used as a recipient genotype for *Agrobacterium*-mediated transformation in our laboratory. It was therefore selected for functional characterization because of its breeding relevance and suitability for stable genetic transformation.

The *rlp23-1* mutant carrying a T-DNA insertion in *AtRLP23* (SALK_034225) was obtained from AraShare. The constructs were introduced into Col-0 (for overexpression) or *rlp23-1* (for complementation) plants via *A*. *tumefaciens*-mediated transformation [31].

Transgenic *B. napus* overexpression lines were generated through *Agrobacterium*-mediated hypocotyl transformation followed by tissue culture regeneration [32]. Putative transformants were selected by antibiotic resistance screening and subsequently confirmed by PCR analysis.

*S. sclerotiorum* strain 1980 (wild-type) was routinely cultured on potato dextrose agar (PDA) plates at 20 °C for routine propagation and preserved at 4 °C for extended storage.

### 4.6. ROS Burst Measurement Assay

The ROS production assay was conducted according to a previously established protocol with minor modifications [34]. Leaf discs were collected from fully expanded leaves of soil-cultivated *A. thaliana* plants (five-week-old) and placed in 96-well plates filled with 200 μL sterile ddH_2_O per well, followed by overnight incubation in darkness. Before detection, each well was supplemented with 170 μL of elicitation mixture consisting of 1 μM Ssnlp24_SsNEP2_ peptides, 20 μg/mL horseradish peroxidase (HRP), and 100 μM luminol. Chemiluminescence signals were monitored every 2 min for approximately 60 min using a Spark^®^ microplate reader (Tecan, Männedorf, Switzerland).

### 4.7. S. sclerotiorum Infection Assay

Mycelial discs of *S. sclerotiorum* (wild-type strain), with diameters of 2 or 5 mm, were obtained from the margins of actively expanding colonies grown on PDA plates for 48 h. These fungal discs were subsequently transferred onto detached leaves. *A. thaliana* seedlings (four-week-old) and *B. napus* plants (120-day-old) were selected for pathogen inoculation experiments. Lesion areas were measured at 36 or 48 h post-inoculation and quantified using ImageJ (version 1.0). For each genotype, three leaves were used for each treatment, with three independent biological experiments performed. At least two independent overexpression lines were obtained for each of BnaRLP-G12-2/3/4. Two independent transgenic lines were selected for pathogen infection assays in Arabidopsis, whereas the line exhibiting the highest transcript abundance was selected for infection assays in *B. napus*.

### 4.8. RT-qPCR Analysis

The expression levels of *BnaRLP-G13-1/2/3/4* were analyzed following *S. sclerotiorum* infection or Ssnlp24_SsNEP2_ treatment. For pathogen infection, leaf samples of *B. napus* (XY15) were collected at 0, 24, 48, and 96 h following inoculation with the wild-type strain of *S. sclerotiorum*. The collected tissues comprised a 5 mm region adjacent to the inoculation point, encompassing both infected areas and visible necrotic lesions. For peptide treatment, fully expanded leaves of XY15 were treated with 1 μM Ssnlp24_SsNEP2_, and tissue samples were harvested at 4 and 24 h post-treatment. Specific primers were designed using Primer Premier 5.0 software, and the resulting PCR products ranged from 100 to 400 bp in length (Supplementary Table S2). Relative transcript abundance was determined using the comparative 2^−ΔΔCt^ method. The internal reference was *BnaUBC9* [45]. The detailed information on reagents is provided in Supplementary Table S4.

### 4.9. Subcellular Localization Assay

The coding regions of BnaRLP-G13-2/3/4 without stop codons were amplified and cloned into the pCAMBIA1300-35S-eGFP vector using the SacI and SalI restriction sites, generating C-terminal eGFP fusion constructs. The empty eGFP vector was used as a control. The plasma membrane marker AtAtPIP2A-mCherry was used for co-localization analysis. All constructs were introduced into *A. tumefaciens* GV3101. The subsequent agroinfiltration and fluorescence detection procedures were performed as described above for the BiFC assays. GFP fluorescence was detected with excitation/emission wavelengths of 470–490/500–550 nm, while mCherry fluorescence was monitored at 575–595/580–630 nm.

### 4.10. Statistical Analysis

All experiments were performed with three biological replicates, and the data were pooled for statistical analysis. Data are presented as mean ± standard deviation (SD). The expression levels of *BnaRLP-G13* members following *S. sclerotiorum* infection or Ssnlp24_SsNEP2_ treatment were analyzed by two-way ANOVA with Tukey’s test. For lesion area measurements, one-way ANOVA followed by Dunnett’s test was performed to compare transgenic lines with the corresponding wild-type plants under the same growth conditions. SPSS software was used for statistical analyses (version 22.0; IBM Corporation, Armonk, NY, USA).

## Data Availability

Data are contained within the article and Supplementary Materials.

## Supplementary Materials

The following supporting information can be downloaded at: https://www.mdpi.com/article/10.3390/plants15162545/s1, Figure S1: Analysis of interactions between YCE and candidate proteins by BiFC. Figure S2. Alignments of the resequencing sequence of *BnaRLP-G13-2.* Figure S3. Alignments of the resequencing sequence of *BnaRLP-G13-3*. Figure S4. Alignments of the resequencing sequence of *BnaRLP-G13-4*. Figure S5. Secondary structure of BnaRLP-G13-2/3/4. Figure S6. Subcellular localization of BnaRLP-G13-2/3/4 protein. Figure S7. BnaRLP-G13-2/3/4 overexpression in Col-0. (A) Genotype identification of overexpression lines was performed using semi-quantitative PCR of *BnaRLP-G13-2/3/4*. (B) Phenotype of *35S::BnaRLP23*/Col and Col-0. Figure S8. BnaRLP-G13-2/3/4 overexpression in XY15. (A) Relative expression levels of *BnaRLP-G13-2/3/4* in *35S::BnaRLP-G13-2/3/4*/XY15. (B) Phenotype of *35S::BnaRLP-G13-2/3/4*/XY15 and XY15. Figure S9. Genotype identification of the complementation lines was performed using semi-quantitative PCR of *BnaRLP-G13-2/3/4*. Figure S10. Expression of *BnaRLP-G13-1* following Ssnlp24_SsNEP2_ treatment.; Table S1: Basic information and structural features of *BnaRLP-G13* family members in *B. napus.* Table S2. Primers used in this study. Table S3. Bioinformatics tools used for structural analyses. Table S4. Information on reagents used for RT-qPCR analysis.
