# Supplementary material for: Multiple BnaSOBIR1-Associated Receptor-like Proteins Contribute to Enhanced Resistance to Sclerotinia sclerotiorum in Brassica napus L. (Oilseed Rape)
Source: Plants (Basel). 2026 Aug 21;15(16):2545. doi: 10.3390/plants15162545 (PMC13516149; doi:10.3390/plants15162545)
Supplement: Supplementary file 1 [file plants-15-02545-s001.zip › plants-4459944-supplementary.pdf]

Supplementary Information

1 Supplementary Figures

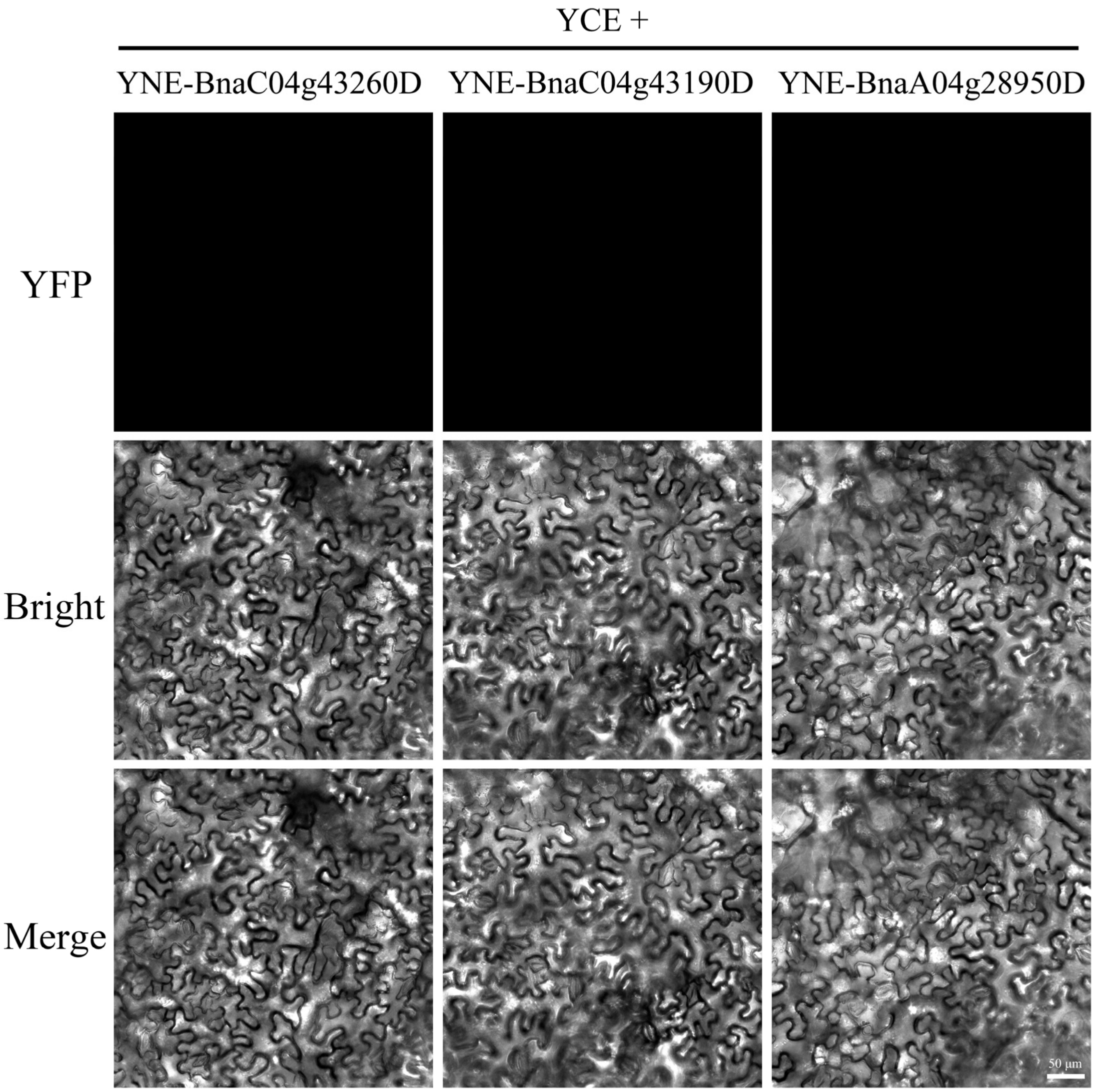

Supplementary Figure S1. Analysis of interactions between YCE and candidate proteins by BiFC. Three candidate proteins (BnaC04g43260D, BnaC04g43190D, and BnaA04g28950D) were fused to YNE. Merged images represent the overlay of YFP fluorescence and bright-field microscopy images.

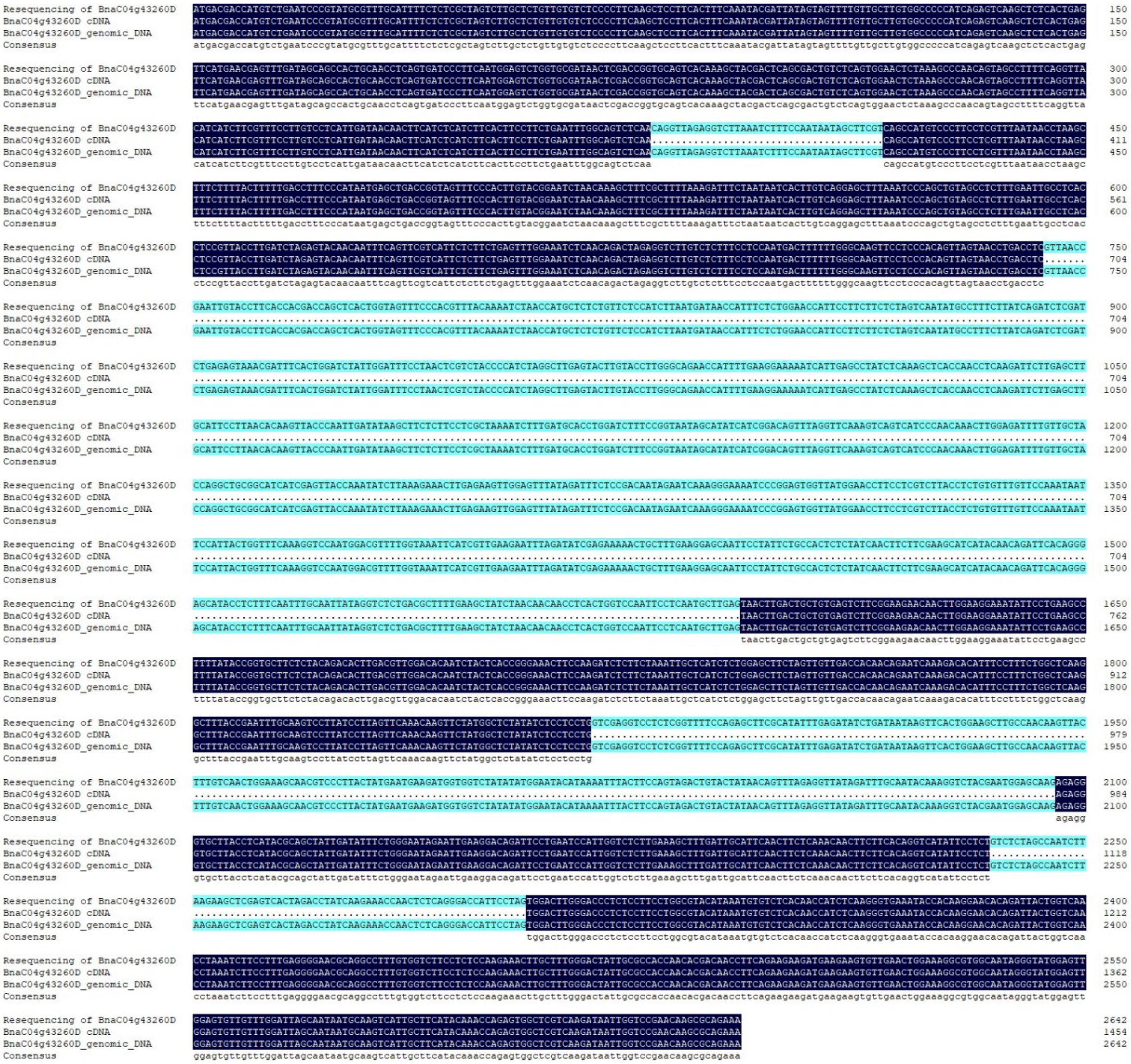

Supplementary Figure S2. Alignments of the resequencing sequence of *BnaRLP-G13-2*. The first row was resequencing results of *BnaRLP-G13-2* (the amplified sample was derived from RNA of XY15), the second row was cDNA sequences of *BnaRLP-G13-2* in the database, and the third row was genomic DNA sequences of *BnaRLP-G13-2* in the database.

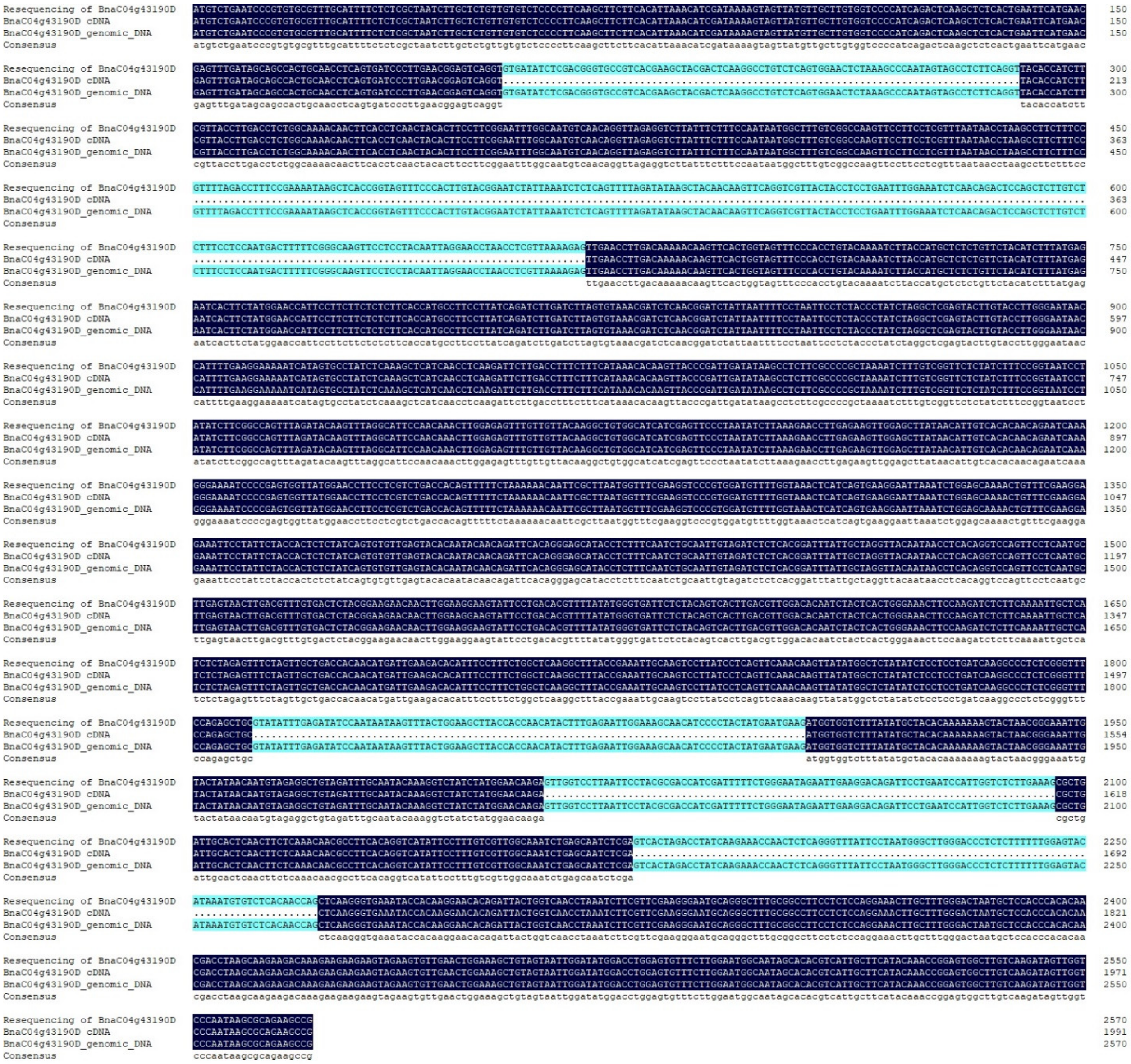

Supplementary Figure S3. Alignments of the resequencing sequence of *BnaRLP-G13-3*. The first row was resequencing results of *BnaRLP-G13-3* (the amplified sample was derived from RNA of XY15), the second row was cDNA sequences of *BnaRLP-G13-3* in the database, and the third row was genomic DNA sequences of *BnaRLP-G13-3* in the database.

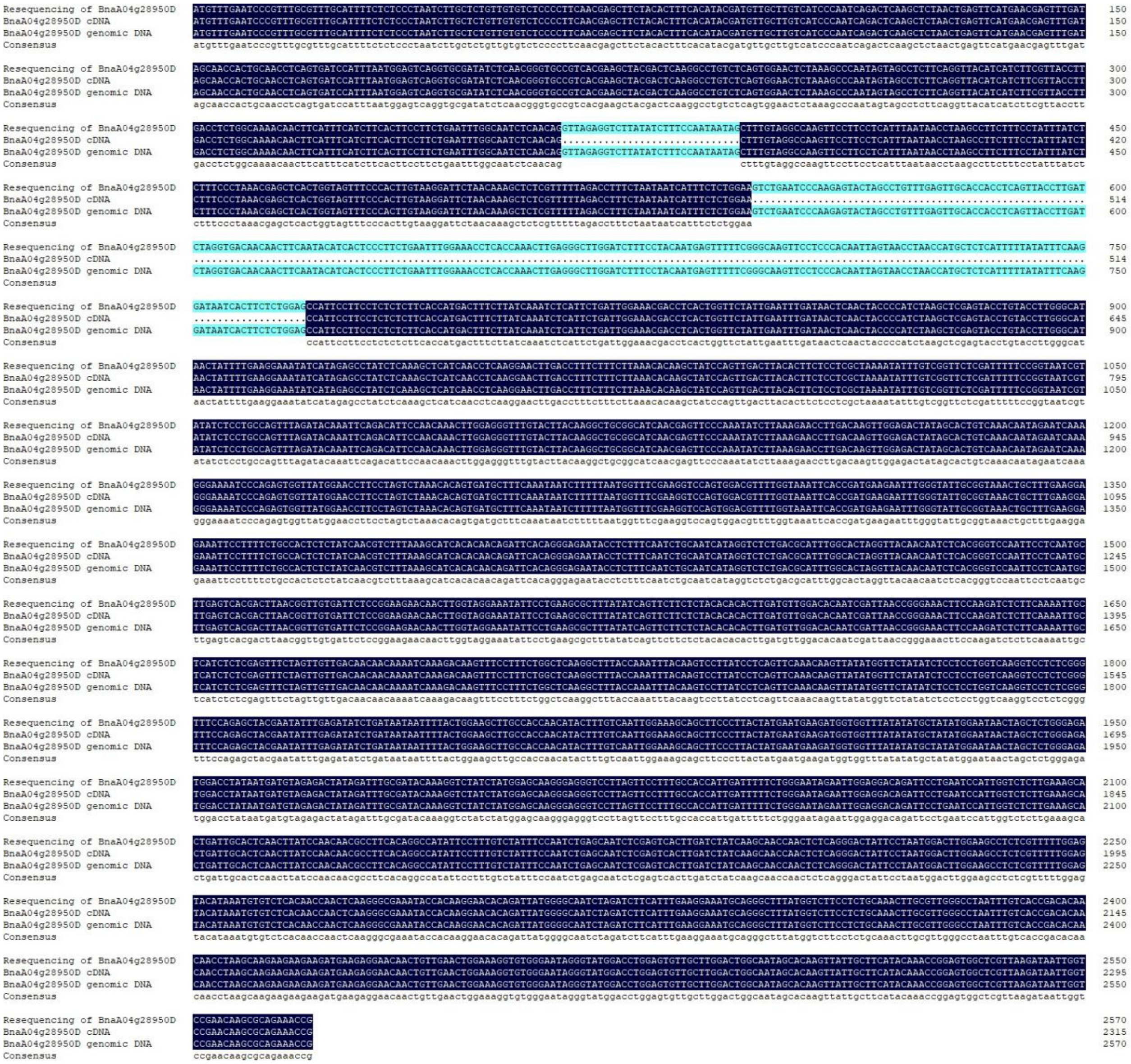

Supplementary Figure S4. Alignments of the resequencing sequence of *BnaRLP-G13-4*. The first row was resequencing results of *BnaRLP-G13-4* (the amplified sample was derived from RNA of XY15), the second row was cDNA sequences of *BnaRLP-G13-4* in the database, and the third row was genomic DNA sequences of *BnaRLP-G13-4* in the database.

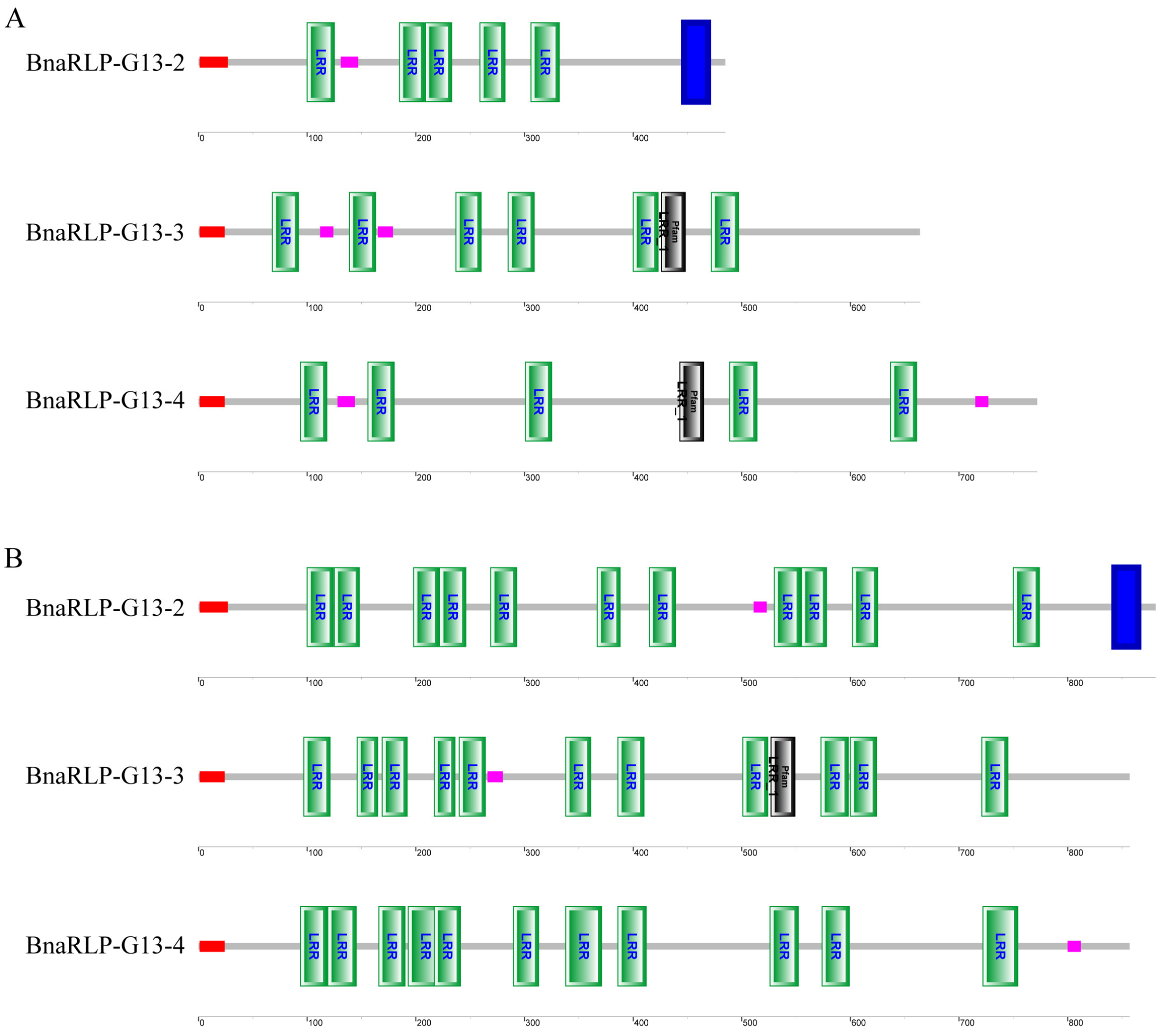

Supplementary Figure S5. Secondary structure of BnaRLP-G13-2/3/4. (A) Prediction results for the BnaRLP-G13-2/3/4 sequence obtained from the BRAD database. (B) Prediction results for the BnaRLP-G13-2/3/4 sequence cloned from XY15 and resequenced. The red boxes indicate the signal peptide, the green boxes represent the leucine-rich repeats (LRR) domain, the blue boxes indicate the transmembrane domain and the purple boxes indicate the low complexity region.

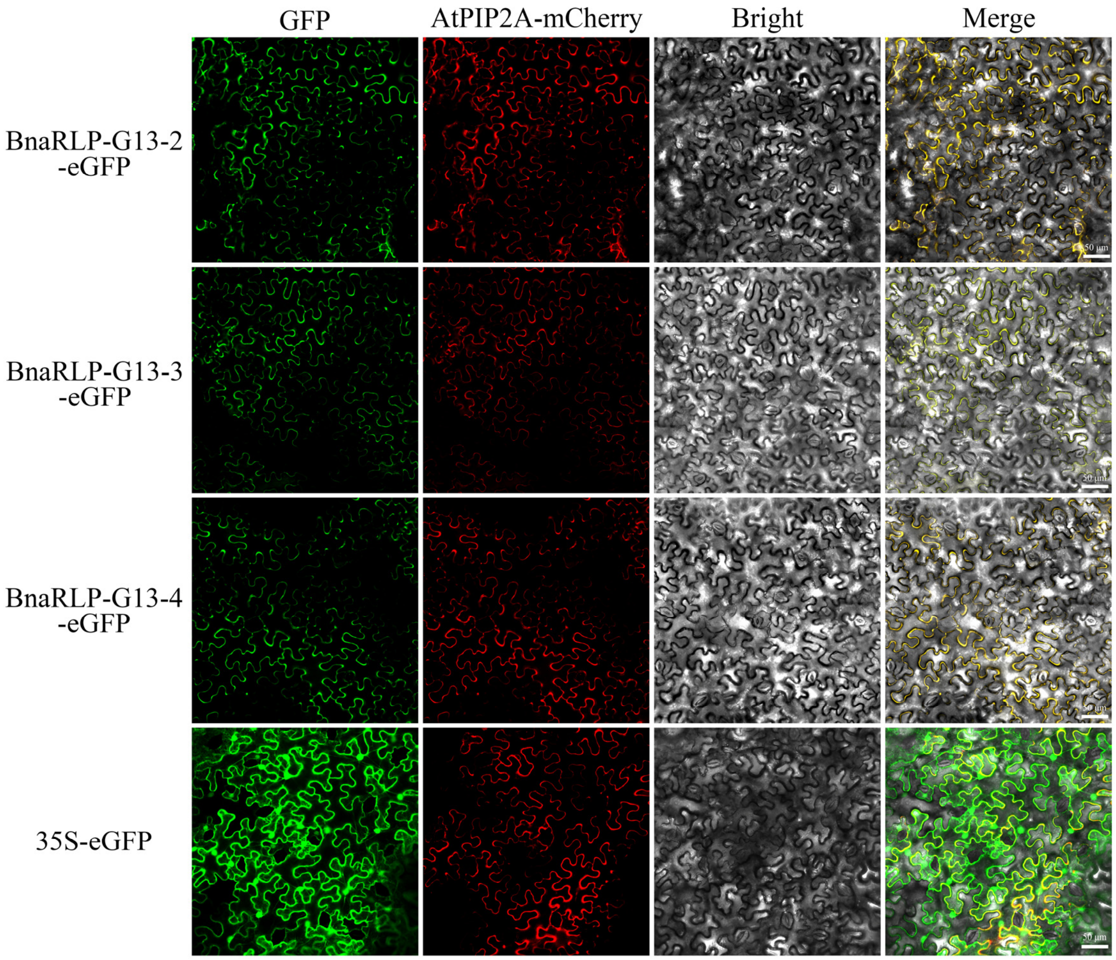

Supplementary Figure S6. Subcellular localization of BnaRLP-G13-2/3/4 protein. BnaRLP-G13-2/3/4-eGFP transiently expressed in *N. benthamiana* leaves with 35S-eGFP construct as negative control. 35S-eGFP vector was used as a control. AtAtPIP2A-mCherry was used as plasma membrane marker. Scale bar = 50 μm. Merge: Merged via GFP, mCherry and bright-field microscopy.

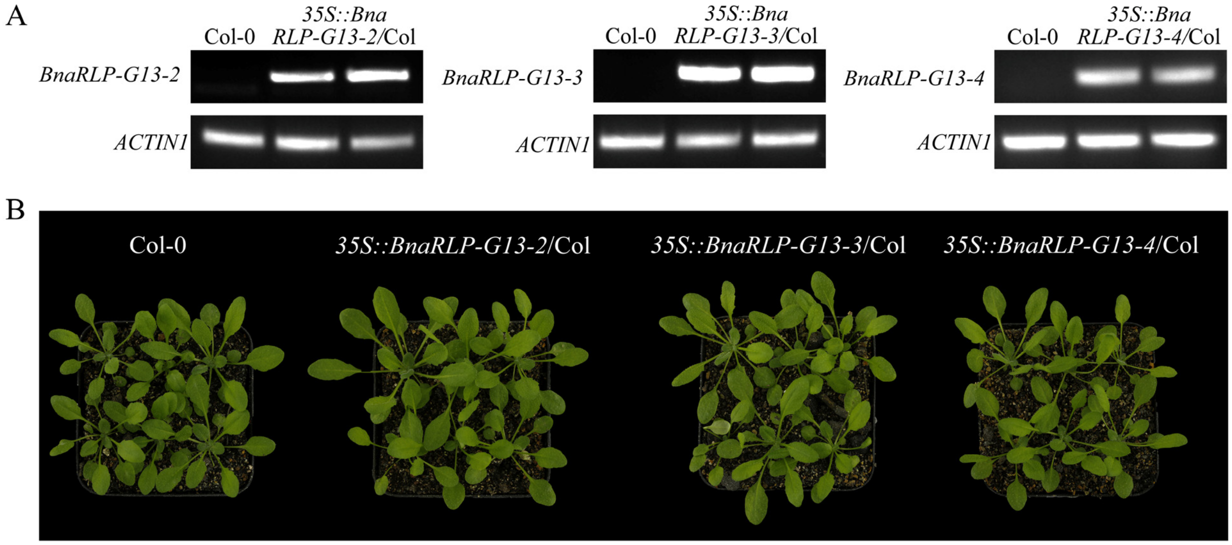

Supplementary Figure S7. *BnaRLP-G13-2/3/4* overexpression in Col-0. (A) Genotype identification of overexpression lines was performed using semi-quantitative PCR of *BnaRLP-G13-2/3/4*, *ACTIN1* as the reference gene. (B) Phenotype of *35S::BnaRLP23*/Col and Col-0, all plants were grown on soil at 22°C in parallel and photographed when they were 4 weeks old.

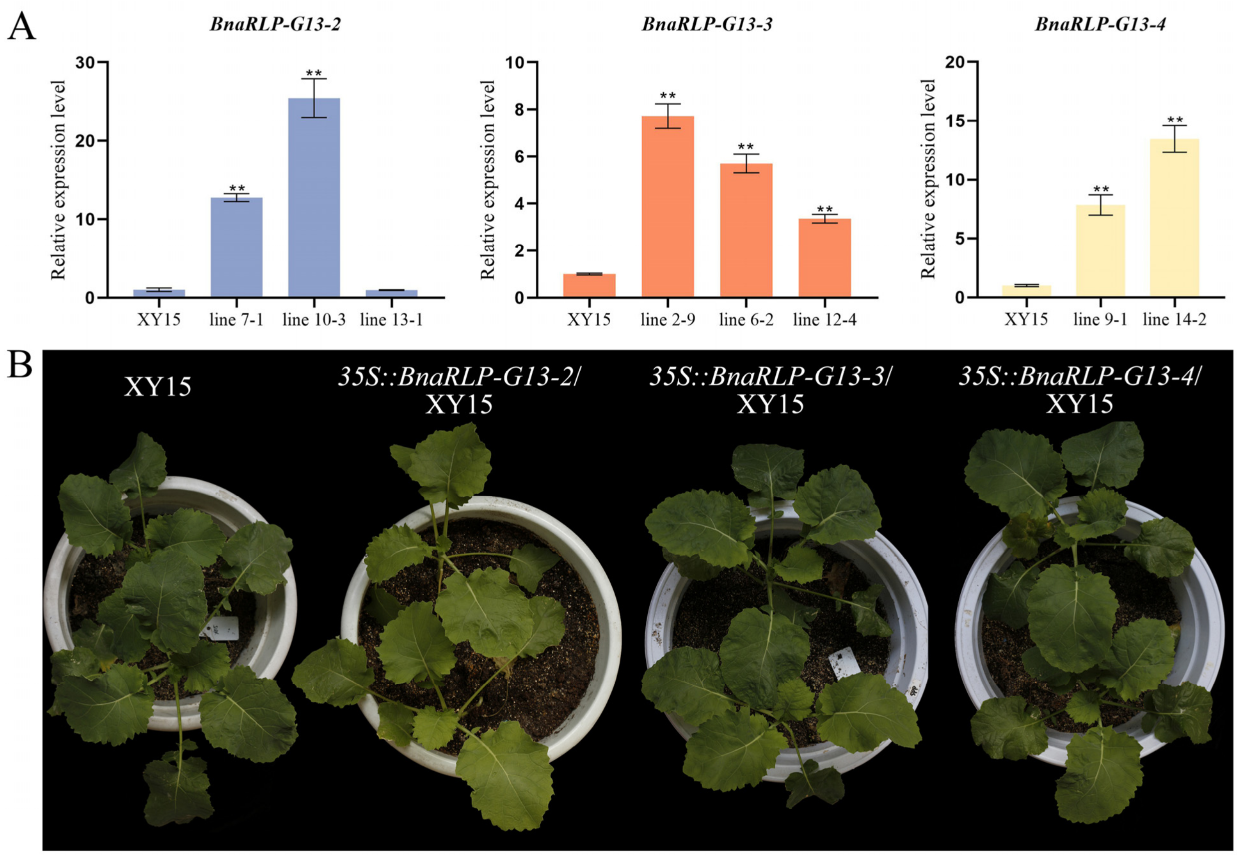

Supplementary Figure S8. *BnaRLP-G13-2/3/4* overexpression in XY15. (A) Relative expression levels of *BnaRLP-G13-2/3/4* in *35S::BnaRLP-G13-2/3/4*/XY15. Expression levels were normalized to *BnaUBC9*. Error bars indicate standard deviation (SD). Statis-tical significance was determined using Student's t-test (**$p < 0.01$). (B) Phenotype of *35S::BnaRLP-G13-2/3/4*/XY15 and XY15, all plants were grown on soil at 22°C in parallel and photographed when they were 120-days old.

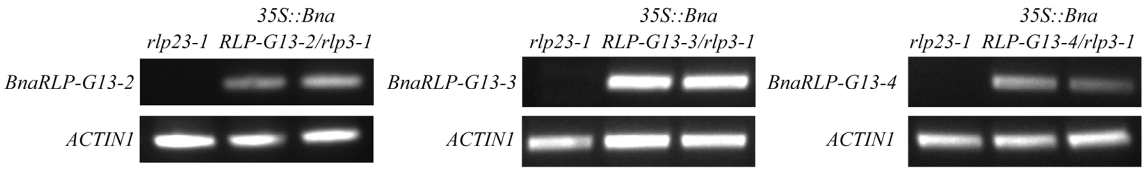

Supplementary Figure S9. Genotype identification of the complementation lines was performed using semi-quantitative PCR of BnaRLP-G13-2/3/4. ACTIN1 as the reference gene.

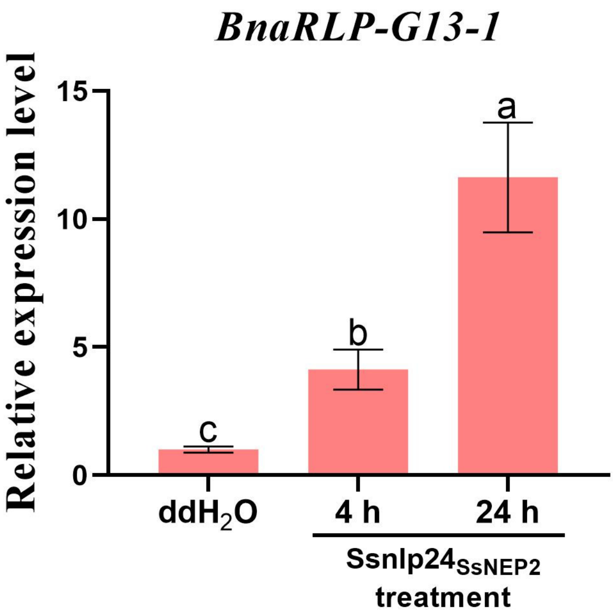

Supplementary Figure S10. Expression of *BnaRLP-G13-1* following Ssnlp24$_{SsNEP2}$ treatment. Relative expression levels of *BnaRLP-G13-1* in XY15 following treatment with 1 μM Ssnlp24$_{SsNEP2}$ at 4 and 24 h, as determined by RT-qPCR. Expression levels were normalized to *BnaUBC9*. Error bars indicate standard deviation (SD). Statistical significance was determined using Student's t-test. Asterisks indicate significant differences compared with the ddH$_2$O-treated control (** $p < 0.01$).

2 Supplementary Tables

Supplementary Table S1. Basic information and structural features of *BnaRLP-G13* family members in *B. napus.*

Gene name	Database ID	Chromosome location	Protein length (aa)	Molecular weight (kDa)	Signal peptide	Transmembrane domain
BnaRLP-G13-1	*BnaC04g56380D*	C04	880	97.87	Present	Absent
BnaRLP-G13-2	*BnaC04g43190D*	C04	881	97.73	Present	Present
BnaRLP-G13-3	*BnaC04g43260D*	C04	857	94.94	Present	Absent
BnaRLP-G13-4	*BnaA04g28950D*	A04	857	94.96	Present	Absent

Supplementary Table S2. Primers used in this study.

Primer name	Primer sequence	Primer function
14760-STE-F	tctattttatgtaatggccattacggccatgattacaatggctgt	Y2H library screening
14760-STE-R	cgaattcctgcagatggccgaggcggccccgtgtttgatctgggaca	
18590-STE-F	tctattttatgtaatggccattacggccatggctgctcccaccggaaa	
18590-STE-R	cgaattcctgcagatggccgaggcggccccgtgcttgatctgggacag-cataat	
pPR3-N F	gtcgaaaattcaagacaagg	
pPR3-N R	aagcgtgacataactaatta	
YNE-G13-2-F	acgggggactctagaggtaccatgacgaccatgtctga	BiFC
YNE-G13-2-R	ccatgtcgacactagtggatccatttctgcgcttgttcgga	
YNE-G13-3-F	cacgggggactctagaggtaccatgtctgaatcccgtgtgc	
YNE-G13-3-R	ccatgtcgacactagtggatccacggcttctgcgcttattg	
YNE-G13-4-F	acgggggactctagaggtaccatgtttgaatcccgtttgc	
YNE-G13-4-R	catgtcgacactagtggatccacggtttctgcgcttgttcg	
YCE-SOBIR1-1-F	ggagagaacactgcagggtaccatgattacaatggctgt	
YCE-SOBIR1-1-R	acatgtcgacactagtggatccgtgtttgatctgggaca	
YCE- SOBIR1-2-F	ggagagaacactgcagggtaccatggctgctcccaccggaaac	
YCE- SOBIR1-2-R	acatgtcgacactagtggatccgtgcttgatctgggacagcataat	
YCE-nlp24-F	gagagaacactgcagggtaccatgggactgatgtattcc	
YCE-nlp24-R	acatgtcgacactagtggatccttcccagtcgtggcg	
G13-2-STE-F	tctattttatgtaatggccattacggccatgacgaccatgtctga	Y2H
G13-2-STE-R	cgaattcctgcagatggccgaggcggccccatttctgcgcttgttcgga	
G13-3-STE-F	tctattttatgtaatggccattacggccatgtctgaatcccgtgtgc	
G13-3-STE-R	cgaattcctgcagatggccgaggcggccccacggcttctgcgcttattg	
G13-4-STE-F	tctattttatgtaatggccattacggccatgtttgaatcccgtttgc	
G13-4-STE-R	cgaattcctgcagatggccgaggcggccccacggtttctgcgcttgttcg	
pPR3N-14760-F	ggtatcaacgcagagtggccattacggccatgattacaatggctgt	

Primer name	Primer sequence	Primer function
pPR3N-14760-R	tatcgaattctcgagaggccgaggcggccctagtgtttgatctgggaca	
pPR3N-18590-F	ggtatcaacgcagagtggccattacggccatggctgctcccaccggaaa	
pPR3N-18590-R	cgaattctcgagaggccgaggcggccctagtgcttgatctgggacagca	
G13-1-qF	ggagggtccttacttcatacgc	RT-qPCR
G13-1-qR	taggttgccccgttatttgcg	
G13-2-qF	cccttcaatggagtctggtg	
G13-2-qR	ggtaacggaggtgaggcaat	
G13-3-qF	cggtaatcctatatcttcggc	
G13-3-qR	aacatccacgggaccttcg	
G13-4-qF	gggtattgcggtaaactgc	
G13-4-qR	tagtgccaaatgcgtcagag	
UBC9-qF	gcatctgcctcgacatcttga	
UBC9-qR	gacagcagcaccttggaaatg	
35S-G13-2-F	gagaacacgggggactggtaccatgacgaccatgtctga	Overexpression and complementation
35S-G13-2-R	gcaggtcgactctagaggatccatttctgcgcttgttcgg	
35S-G13-3-F	gagaacacgggggactggtaccatgtctgaatcccgtgtgc	
35S-G13-3-R	gcaggtcgactctagaggatccacggcttctgcgcttattg	
35S-G13-4-F	gagaacacgggggactggtaccatgtttgaatcccgtttg	
35S-G13-4-R	gcaggtcgactctagaggatccacggtttctgcgcttgttcg	
G13-2-eGFP-F	gagaacacgggggacgagctcatgacgaccatgtctga	Subcellular Localization
G13-2-eGFP-R	ccttgctcaccatggtgtcgactttctgcgcttgttcgg	
G13-3-eGFP-F	gagaacacgggggacgagctcatgtctgaatcccgtgtgc	
G13-3-eGFP-R	ccttgctcaccatggtgtcgaccggcttctgcgcttattg	
G13-4-eGFP-F	gagaacacgggggacgagctcatgtttgaatcccgtttg	
G13-4-eGFP-R	ccttgctcaccatggtgtcgaccggtttctgcgcttgttcg	

Supplementary Table S3. Bioinformatics tools used for structural analyses.

Software/Database	Website
Gene Structure Display Server (GSDS) 2.0	http://gsds.cbi.pku.edu.cn/
MEME Suite	http://meme-suite.org/tools/meme
SignalP 5.0	http://www.cbs.dtu.dk/services/SignalP/
SMART	http://smart.embl-heidelberg.de/
TMHMM Server v2.0	http://www.cbs.dtu.dk/services/TMHMM/
Pfam database	http://pfam.xfam.org/

Supplementary Table S4. Information of reagents used for RT-qPCR analysis.

Step	Details
RNA extraction	Total RNA was extracted using the Eastep™ Super Total RNA Extraction Kit.
cDNA preparation	First-strand cDNA was generated with the GoScript™ Reverse Transcription System Kit (Promega, USA).
qPCR reaction	RT-qPCR was performed using SYBR® Green Premix Pro Taq HS qPCR Kit II (AG11702, Accurate Biotechnology, China) on a StepOne™ Real-Time PCR System.
